# Evidence for a CO_2_ ‐concentrating mechanism in the model streptophyte green alga *Chara braunii*

**DOI:** 10.1111/nph.70283

**Published:** 2025-06-13

**Authors:** Carolin M. Heise, Daniel A. Heß, Peter Walke, Maren Voß, Hendrik Schubert, Wolfgang R. Hess, Martin Hagemann

**Affiliations:** ^1^ Plant Physiology Department Faculty of Mathematics and Natural Sciences, University of Rostock Einsteinstr. 3 18059 Rostock Germany; ^2^ Aquatic Ecology Department Faculty of Mathematics and Natural Sciences, University of Rostock Einsteinstr. 3 18059 Rostock Germany; ^3^ Genetics and Experimental Bioinformatics Group Faculty of Biology, University of Freiburg Schänzlestr. 1 79104 Freiburg Germany; ^4^ Marine Nitrogen Cycle, Department of Biological Oceanography Leibniz‐Institute for Baltic Sea Research Warnemünde (IOW) 18119 Rostock Germany

**Keywords:** bicarbonate, carbonic anhydrase, Charophyceae, CO_2_ limitation, gene expression, transport

## Abstract

- Oxygenic photosynthesis in streptophytic algae, such as Charophyceae, is often impeded by low CO_2_ levels in aquatic habitats. Consequently, many algal groups evolved a CO_2_‐concentrating mechanism (CCM). However, its presence in Charophyceae remains controversial.
- To explore this, we analyzed the acclimation of photosynthesis, carbon isotope composition, and gene expression in *Chara braunii* under varying inorganic carbon (Ci) conditions.
- The photosynthetic activity changed complementarily under low‐ or high‐Ci levels. Notably, the Ci compensation point of photosynthesis was significantly lower in thalli grown at ambient Ci than in elevated Ci. Correspondingly, the delta ^13^C levels were lower in thalli from high than low Ci. These results indicate that *C. braunii* performs a CCM under low Ci, which is suppressed under high Ci. Transcriptomic analyses of algae from different Ci cultivations provided insight into Ci‐regulated genes and pointed to the possible association between carbonic anhydrases and aquaporins with the CCM.
- Collectively, our results indicate that *C. braunii* expresses a CCM allowing efficient use of CO_2_ and bicarbonate under limiting Ci conditions. A tentative scenario is provided summarizing the role of potential players in the CCM.

## Introduction

Charophyceae form a class of multicellular green algae that belongs to the Streptophyta clade, which also includes all terrestrial plants (Wodniok *et al*., 2011; de Vries & Archibald, 2018; Hess *et al*., 2022). They are the morphologically most complex streptophyte algae, exhibiting features thought to be essential for successful colonization of the terrestrial habitat (e.g. functional rhizoids, gametangia, envelopes), and were considered early as a sister lineage of land plants (e.g. Pringsheim, 1863). During evolution, Charophyceae adapted to a wide range of environmental conditions, and some species can withstand rather extreme conditions. For example, they can thrive under acidic as well as alkaline conditions and cover the full range of ultraoligotrophic to hypertrophic conditions in waters of different salinities (Schubert *et al*., 2017, 2018). Supported by the currently available draft genome sequence (Nishiyama *et al*., 2018), *Chara braunii* has become a model system for streptophyte and early land plant evolution (Kurtović *et al*., 2023), also permitting systematic analyses of the occurrence and evolution of CO_2_‐concentrating mechanisms (CCMs) among Charophyceae.

All organisms performing oxygenic photosynthesis fix CO_2_ in the Calvin–Benson–Bassham cycle using ribulose‐bisphosphate carboxylase/oxygenase (RubisCO). As aquatic organisms, Charophyceae must cope with low‐CO_2_ solubility in water, which can limit the CO_2_ fixation by RubisCO. Moreover, the total pool of inorganic carbon (Ci = inorganic carbon; CO_2_, bicarbonate, and carbonate) is highly variable in this environment. Its amount and composition depend on the pH, temperature, and salinity, which influence the solubility of CO_2_ and its conversion to bicarbonate and at high pH into carbonate (Kupriyanova *et al*., 2023). Many algae use bicarbonate, the dominant source of Ci under alkaline conditions, which can be accumulated via active transport mechanisms, whereas land plants rely solely on gaseous CO_2_ that diffuses into photosynthetic tissues and cells. The available pCO_2_ relative to pO_2_ has a large impact on photosynthetic CO_2_ fixation via type 1 RubisCO, which is characterized by low‐CO_2_ affinity and low discrimination between CO_2_ and O_2_ (Tcherkez *et al*., 2006; Hagemann *et al*., 2016). Therefore, many eukaryotic algae employ sophisticated CCMs to enrich CO_2_ in the vicinity of RubisCO, which results in a higher Ci affinity of the organism than the Km_CO2_ of its RubisCO. However, the existence and function of a CCM in Charophytes is uncertain (Raven *et al*., 2012) but has been demonstrated in streptophytic green algae Zygnematophyceae, the sister group of land plants (Goudet *et al*., 2020).

Among eukaryotic algae, the CCM is best understood in the model chlorophyte *Chlamydomonas reinhardtii* and the diatom *Phaeodactylum tricornutum* (Wang *et al*., 2015; Matsuda *et al*., 2017; Mackinder, 2018). In these organisms, RubisCO is confined to the pyrenoid, a structure visible in electron microscope images of the chloroplasts of many algae. There, CO_2_ is enriched due to the combined action of different types of bicarbonate transporters and carbonic anhydrases (CAs), which accumulate Ci at significantly higher levels inside cells than in the surrounding medium. In addition to the so‐called biophysical CCM (mostly relying on transport activities) of algae, C4 plants developed a biochemical CCM, in which CO_2_ is first concentrated in C4 acids (oxaloacetate, malate) due to the action of PEP carboxylase. Subsequently, CO_2_ is released in the vicinity of RubisCO by different decarboxylating enzymes such as NADP‐ or NAD‐specific malic enzymes or PEP carboxykinases (e.g. Pardo & VanBuren, 2021; Clapero *et al*., 2024).

Compared with the CCM in other algae and C4 plants, the Ci acquisition strategies of Charophyceae are not well‐understood. Cytological studies failed to detect pyrenoid‐like structures in the chloroplasts of *Chara* spp. (Pickett‐Heaps, 1975). Many researchers assume that the characteristic banding in alkaline and acidic zones of internodal *Chara* cells may be involved in Ci acquisition. According to these propositions, acidic zones can promote the conversion of bicarbonate into CO_2_ and its efficient use for photosynthesis. The alkaline bands appear as a consequence of exported OH^−^ released during CO_2_ fixation or different activities of H^+^/OH^−^ channels (Schmölzer *et al*., 2011; Beilby & Casanova, 2014; Absolonova *et al*., 2018) and may represent the site for bicarbonate uptake. Electrochemical studies indicated that bicarbonate in the medium has a marked influence on light‐induced membrane polarization effects, which were interpreted as an electroneutral bicarbonate/H^+^ antiport that occurs at internodal cells of *Chara corallina* (Lucas, 1982). Cotransport of bicarbonate and other ions has also been assumed in earlier studies (Raven & Smith, 1982). However, the exact nature of the Ci uptake mechanism of Charophytes is poorly understood (Raven *et al*., 2012).

In this study, *C. braunii* thalli were grown under different Ci conditions. Photosynthetic parameters indicated that the alga performed better under Ci‐enriched conditions. Photosynthetic CO_2_ uptake showed a lower CO_2_ compensation point for *C. braunii* grown under ambient conditions than under elevated Ci conditions. This can be taken as an indication of CCM activity, which was supported by a lowered ^13^C discrimination in thalli cultured at ambient Ci. Transcriptomic analyses identified a specific set of genes that responded differentially to high‐ and low‐Ci levels. Among them, genes for CAs, aquaporins, PEP carboxylases, and PEP carboxykinases showed clear low‐Ci‐induced increases in expression. The obtained data allow us to conclude that *C. braunii* can acclimate to fluctuating Ci conditions by differential expression of a CCM.

## Materials and Methods

### Chara cultivation

The freshwater streptophyte alga *Chara braunii* (Gmelin) S276 was cultivated as described by Holzhausen *et al*. (2022). Double‐autoclaved deionized water was used as the standard cultivation medium on a layer of double‐autoclaved quartz sand, lime, and compost. Passive diffusion of the solid phase of the cultivation setup likely led to low ion concentrations in the liquid medium. Cultivation is described in more detail by Heß *et al*. (2023a). Cultivation under ambient air conditions (0.04% CO_2_) was defined as a low‐inorganic carbon (LC) condition.

### Acclimation to different inorganic carbon (Ci) conditions and sampling

**Fig. 1:**
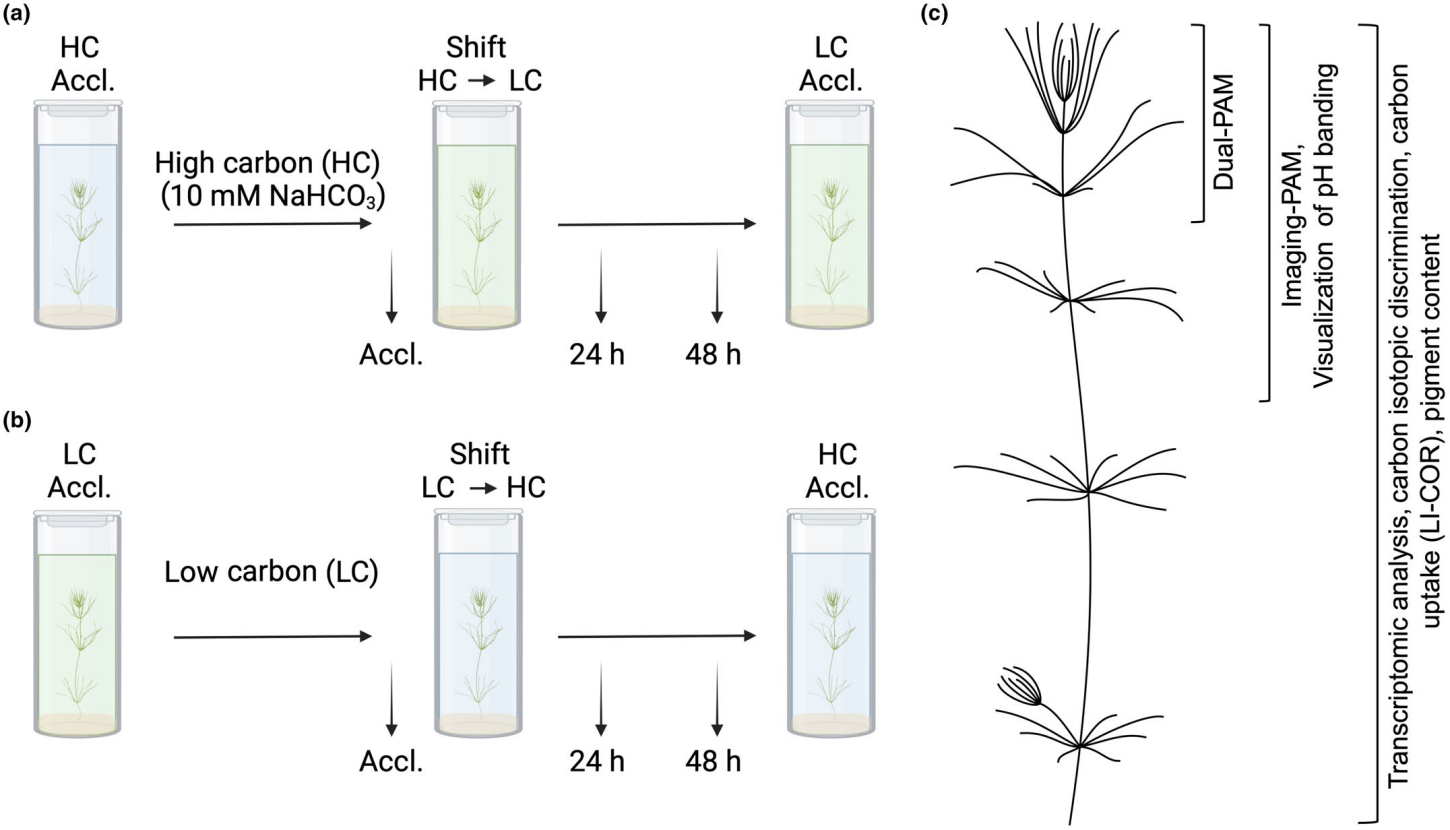
Schematic representation of the cultivation and experimental setup. (a) Visualization of high carbon (HC, 10 mM NaHCO_3_) to low carbon (LC, ambient air) shift of *Chara braunii* cultures. Sampling was performed after the cultures were acclimated to the HC conditions (Accl., after 7 d) and during the HC to LC shift after 24 and 48 h. (b) Schematic visualization of the LC to HC shift with the respective sampling times for acclimated (Accl.) and shifted (24 and 48 h) samples. (a) and (b) were created using BioRender.com (https://BioRender.com/k14u067). The color of the respective medium refers to the conditions: HC is shown in light blue, LC in green. (c) Visualization of algal thalli parts used in the respective analyses.

To evaluate the physiological response of *C. braunii* to fluctuating Ci conditions, the cultivation medium of the algae (LC) was exchanged with medium containing high‐inorganic carbon (HC, 10 mM NaHCO_3_ final concentration). After 7 d, the liquid medium within the cultivation vessel of long‐term LC‐ or HC‐acclimated algae (Accl. LC and Accl. HC, respectively) was exchanged with the respective other medium (HC to LC and LC to HC; Fig. 1) without removing the algae from the sediment layer. Samples were collected 24 and 48 h after the shift. Notably, the culture vessels of the HC to LC shift were carefully rinsed twice with LC medium to avoid leftover excess NaHCO_3_ solution. In addition, *C. braunii* was cultivated under 2% CO_2_ aeration at 21°C with a light intensity of 30–50 μmol photons m^−2^ s^−1^ and a 16 h : 8 h, light : dark cycle for up to 21 d. Acclimated algal samples were harvested for carbon uptake measurements, carbon isotope analysis, and visualization of pH banding (Fig. 1c). Algae exposed to shifting Ci availability were used for RNA extraction, transcriptomic analysis, or Chl fluorescence measurements. Samples for RNA extraction were promptly frozen in liquid nitrogen and stored at −80°C for later processing.

### Photosynthesis: Ci‐dependent measurements

Carbon assimilation was measured with the LI‐6800 Aquatic Chamber (LI‐COR) similar to what was described by Steensma *et al*. (2023). Measurements of preacclimated algae (HC or LC) were performed in 10 ml filtered (0.45 μm) culture medium and in 10 ml pH Tris‐buffered ion solution (50 mM Tris, 2 mM NaCl, 2 mM KCl, and 2 mM CaCl_2_; pH 7). The algae were exposed to different Ci concentrations by bubbling air with defined CO_2_ concentrations through the aquatic chamber attached to the LI‐6800 infrared gas analyzers. This avoided the formation of significant diffusion boundary layers enveloping the thalli.

The solubility of CO_2_ was experimentally determined for both respective media and is included in the data evaluation. At the beginning of the experiment, filtered medium was equilibrated to zero CO_2_. While logging every second for 10 min, CO_2_ inflow was adjusted to the defined concentrations. Then, we calculated the CO_2_ uptake (Flux*, H_2_O‐corrected difference of CO_2_ inflow and outflow) for each time point until the new equilibrium. The overall integral of CO_2_ uptake represents the dissolved CO_2_/inorganic carbon (Ci = dissolved inorganic carbon, DIC = total inorganic carbon TIC). This was achieved by summing up all the measured fluxes at each concentration of CO_2_ aeration (Supporting Information Fig. S1).

In preliminary experiments, we found that the determined concentrations were not affected by the addition of CA or alteration of total airflow. The pH of the medium was monitored before and after the measurements using a pH meter (HQ11D digital pH meter, Hach). The algal photosynthetic response to different CO_2_ levels (0, 50, 100, 200, 400, 800, and 1600 μmol CO_2_ mol^−1^) was then measured at a saturating light intensity of 1000 μmol photons m^−2^ s^−1^.

The difference in oxygen sensitivity between HC‐ and LC‐acclimated algae was determined using the LI‐6800 Aquatic Chamber (LI‐COR). Carbon assimilation at ambient CO_2_ (400 ppm) and saturating light intensity of 1000 μmol photons m^−2^ s^−1^ was measured while supplying 3%, 21%, or 40% of O_2_ by an external oxygen mixing device (balanced with N_2_; GMS600_2CH; QCAL Messtechnik GmbH, Munich, Germany). Connection and airflow were set up following the manufacturer's instructions (LI‐COR).

### Photosynthesis: dual‐PAM measurements (Chl fluorescence)

Chl fluorescence was measured by pulse amplitude modulation (PAM) using a DUAL‐PAM‐100 (Walz) at room temperature as described (Heß *et al*., 2023a). Measurements were taken on the youngest apical whorls after dark‐adapting the algae for 20 min and quickly rinsing the cut whorls in LC medium. Measurements were taken in a quartz cuvette containing 1 ml of deionized water supplemented with 2 mM NaCl, 2 mM KCl, and 2 mM CaCl_2_. Since our initial hypothesis was that anion transporters might be involved in Ci acquisition, these salt additions eliminated the possibility that ion transport was limited due to a lack of ion availability. These data were evaluated as described by Heise *et al*. (2024). Photosynthetic pigments were quantified after extraction in 3 ml of N, N‐dimethylformamide, as described by Heß *et al*. (2023a).

### Carbon isotopic discrimination of alga thalli

Isotopic discrimination values of ^13^C were determined from pooled *C. braunii* thalli in four biological replicates. Measurements were taken with two technical replicates each. Excess water was removed by gently tapping the algae on filter paper, and then, the samples were frozen in liquid nitrogen and freeze‐dried. After sampling the algal material, the respective culture medium was removed from the culture vessel, filtered through 55‐μm gauze, and Ci was precipitated with Ba(OH)_2_ for 3 h in sealed beakers. Approximately 0.5 mg of alga thalli and 4.5 mg of carbonate samples were weighed in tin capsules. The isotopic composition was evaluated using an EA Isolink CN Elemental Analyzer coupled via a ConFlo IV Interface to a Delta V Advantage Isotope Ratio Mass Spectrometer (Thermo Fisher Scientific, Dreieich, Germany) relative to the isotopic standard Vienna PeeDee Belemnite. The analytical precision of the stable isotope ratio was < ±0.2‰ (per mil, ‰). Acetanilide (Merck KGaA, Darmstadt, Germany) was used as calibration material. At least two samples were used and measured twice. The obtained ^13^C isotope discrimination of the algal thalli was corrected using the isotope composition of the precipitated carbonate from the medium of the respective culture vessel.

### Imaging PAM and visualization of pH

The pH banding was visualized with phenol red (Carl Roth) of five *C. braunii* thalli cultivated under LC conditions. The indicator solution was prepared as described by the manufacturer; that is, 0.04 g phenol red was dissolved in 1.13 ml NaOH (0.1 mol l^−1^) and filled up to 100 ml with MilliQ water. For the images, the medium was filtered through 0.45‐μM filters, and 1 ml phenol red solution was added. The culture medium was additionally supplemented with 0.1 M HCl until the same color as the buffered medium (Tris 50 mM, pH 7) was obtained. Three whorls were transferred to a microscope slide, excess water was removed with a pipette, and 1 ml of the colored solution was carefully pipetted onto the slide. A tube‐sealing compound (Cha‐seal; Chase Scientific Glass, Rockwood, TN, USA) was used to create a small separation, and a second microscope slide was added on top. The algal samples were dark‐adapted for 10 min and subsequently illuminated (*c*. 180 μmol photons m^−2^ s^−1^) for 10 min to accelerate the formation of the pH‐banding pattern. Images were taken with a Sony Alpha 7 (ILCE‐7) camera.

Subsequently, the samples were immediately transferred to the x‐y stage of the Maxi‐version Imaging PAM Chl fluorometer (Walz, Effeltrich, Germany). Measuring light pulse frequency was set to 1 Hz with an intensity of 4. A saturation pulse was applied (intensity of 5), and *F*_o_ and *F*_m_ were determined in conjunction. Effective photosystem II (PSII) quantum yield (Y(II)) was determined and calculated using the ImagingWin software (v.2.57q39). After the first measurements, the medium was carefully removed with a pipette, and then buffered medium was applied. Algal samples were treated as described previously. Images obtained from the Imaging PAM were depicted in a false color code (ranging from black via red, orange, yellow, green, blue, and violet to purple), which encode numerical values between 0 and 1. Thus, all measured parameters were normalized to values between 0 and 1. Per image, three areas of interest (AOI) were selected in clear distinguishable blue or green banding areas of the youngest fully separable whorl. Within the buffered medium, areas where banding had been previously observed were selected. Statistical analyses of data were performed using R.

### Preparation of total RNA and RNA‐seq analyses

Total RNA of thalli segments was extracted as described by Heß *et al*. (2023a), using a modified acid guanidinium thiocyanate–phenol–chloroform protocol (Chomczynski & Sacchi, 2006) and PGTX buffer (Pinto *et al*., 2009), but excluding Triton X‐100. The concentration and purity of the isolated RNA were measured in a NanoDrop ND‐1000 spectrophotometer (PEQLAB Biotechnologie, Erlangen, Germany). Residual DNA was removed using the Turbo DNase‐free™ Kit (Thermo Fisher Scientific). RNA was recovered using RNA Clean & Concentrator kits (Zymo Research, Freiburg, Germany). RNA integrity was validated using a 5200 Fragment Analyzer System (Agilent, Waldbronn, Germany).

cDNA libraries of poly A+ selected mRNA were constructed and sequenced as a service provided by the Cologne Center for Genomics (CCG). The cDNA pool generated via the Illumina TruSeq stranded protocol (New England Biolabs, Frankfurt, Germany) was paired‐end‐sequenced using 2 × 100 bp read length.

### Bioinformatic analyses

Data analysis was performed on the European instance galaxy server (The Galaxy Community, 2022) following the available guidelines for reference‐based RNA‐seq analysis (Batut *et al*., 2021, 2022). The quality of the raw reads was assessed using FastQC (Andrews, 2010) and MultiQC (Ewels *et al*., 2016). Trimming of adapter and barcode contamination was performed using cutadapt (Martin, 2011) as previously described (Heß *et al*., 2023b).

Mapping and counting of reads against the published *C. braunii* S276 genome (Nishiyama *et al*., 2018) was performed using RNA STAR (Dobin *et al*., 2013). As input, we used cutadapt‐trimmed paired‐end.fastq files (as collection), as well as the published *C. braunii* .fasta files (https://bioinformatics.psb.ugent.be/gdb/Chara_braunii/) as reference genome. The OrcAE gtf gene model was selected to build an index with known, annotated splice junctions. Any settings and parameters not explicitly mentioned were kept within the basic galaxy settings, unless set as previously described (Batut *et al*., 2022; Heß *et al*., 2023b).

Differential gene expression analysis was performed using DESeq2 (Love *et al*., 2014). Genes were considered differentially expressed between experimental time points if an adjusted *P*‐value ≤ 0.05 and log_2_FC ≥ |1| were met. Gene Ontology (GO) analysis of RNA‐seq data was performed using goseq (Galaxy v.1.44.0) (Young *et al*., 2010) and processed using GO‐figure (Reijnders & Waterhouse, 2021; v.1.0.1), R (v.4.3.1) using Rstudio (v.2023.12.1), with R packages readxl (Wickham & Bryan, 2022; v.1.4.3) and ggplot2 (Wickham, 2016; v.3.4.3).

Venn diagrams of differentially expressed genes were generated with the webtool hosted by VIB/Ugent (https://bioinformatics.psb.ugent.be/webtools/Venn/). All RNA‐seq data can be accessed under BioProject accession no. PRJNA965918 (https://www.ncbi.nlm.nih.gov/sra/PRJNA965918). Genes with significantly changed expression (log_2_FC ≥ I 1 I, *P*‐value ≤ 0.05) 24 h after LC to HC shift are provided in Table S1, and 48 h after LC to HC shift in Table S2. Differentially expressed genes 24 and 48 h after HC to LC shift are listed in Tables S3 and S4, while genes with altered expression after long‐term (steady‐state) cultivation of acclimated thalli in HC vs LC are given in Table S5.

### Phylogenetic analyses

A set of proteins was selected based on their sequence similarity to relevant *C. braunii* enzymes and their characterization in model organisms, such as *C. reinhardtii*. Multiple sequence alignments were generated using M‐coffee (Notredame *et al*., 2000; Di Tommaso *et al*., 2011) and analyzed using the Beast 2 software package v.2.7.3 (Bouckaert *et al*., 2019). Calculations were made using standard BEAUTi settings utilizing the tree prior Yule model, substitution model Blosum62, and MCMC chain length of 1e7, with logged parameters at every 1e4 steps. The Tracer v1.7.2 software (Rambaut *et al*., 2018) was used to validate the effective sample sizes (ESS > 200). TreeAnnotator was used to build maximum clade credibility trees using burnIn of 50% and a posterior probability limit of 0.5 for median node heights. The FigTree v1.4.4 software (Rambaut *et al*., 2018) was used to visualize the generated dendrograms.

### Statistical analyses

Statistical analysis of physiological parameters and gene expression changes was performed as described by Heß *et al*. (2023a). All measurements were taken in biological triplicates if not mentioned otherwise; mean values and SD are presented. Significant differences were tested with ANOVA (Type II tests) after normality (Shapiro–Wilk Normality Test), and homogeneity (Levene's Test for Homogeneity of Variance) was confirmed using Rstudio (v.2023.03.0).

## Results

To analyze the dynamic response of *C. braunii* to changes in Ci levels, we performed two complementary experiments (Fig. 1), each with three biological replicates. First, thalli were precultivated for 7 d under ambient conditions at LC and then shifted to HC for 24 and 48 h by exchanging the media to a solution containing 10‐mM bicarbonate. In a second experiment, thalli were preacclimated to HC in the presence of 10‐mM bicarbonate for 7 d before shifting to LC for 24 and 48 h. Subsequently, photosynthesis parameters and transcriptome composition were examined. Furthermore, ^13^C discrimination (δ^13^C) levels were estimated in algal biomass after long‐term acclimation to HC and LC conditions as well as to long‐term CO_2_‐aeration (2% CO_2_).

### Physiological characterization of Ci acclimation

**Fig. 2:**
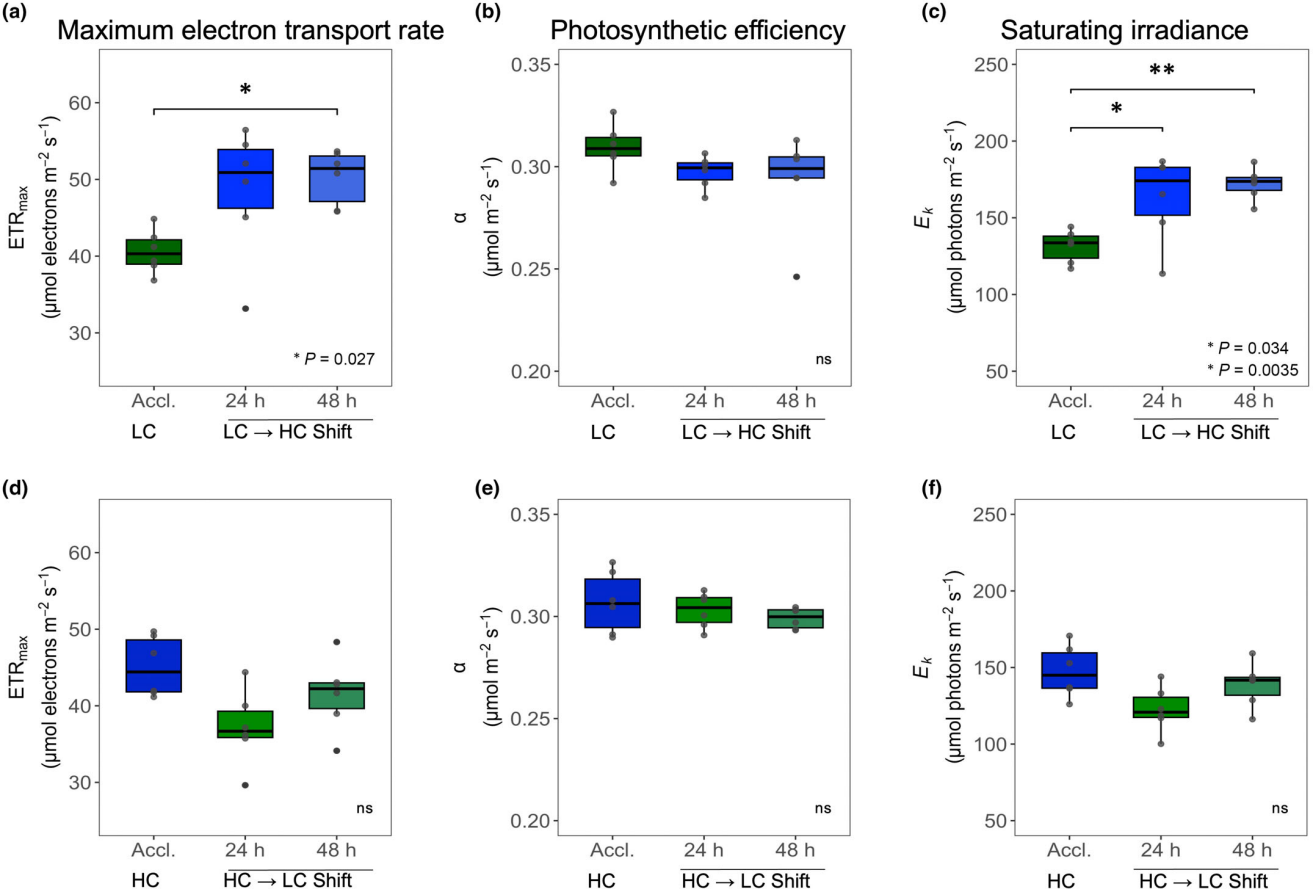
Photosynthetic response of *Chara braunii* after transfer to changing inorganic carbon (Ci) conditions. Box plots show the effect of the Ci regime on photosystem II photosynthetic parameters. These parameters include the maximum electron transport rate (ETR_max_, a, d), the initial slope of the photosynthesis‐irradiance curve (photosynthetic efficiency, α, b, e), and the saturation irradiance point for photosynthesis (*E*_k_, c, f). The upper panel shows the acclimated low‐Ci (Accl. LC) response with the respective shift from LC to high Ci (HC) after 24 and 48 h. The lower panel shows the complementary shift from HC to LC. The color of the box plots corresponds to the respective medium in the cultivation vessel at the time of sampling (blue = HC, green = LC). Box plots of *n* = 6 stretch from the 25^th^ to 75^th^ percentile, black lines inside the boxes indicate the data median, and whiskers represent 1.5× the interquartile range limits. Outliers are shown as black dots and individual data points in gray. *P*‐values were obtained from ANOVA (Type II tests) and Tukey's HSD *post hoc* test (Supporting Information Table S7). Asterisks indicate significantly different mean groups. Differences without statistical significance are not marked, or labeled (ns) at the bottom right corner if applicable for the whole panel.

Photosynthetic activities were first analyzed using fluorescence measurements with the PAM technique. Photosynthesis, as the maximum electron transport rate, was significantly stimulated when thalli were transferred from LC to HC, whereas a nonsignificant decline was observed after shifting thalli from HC to LC (Fig. 2). Similar changes were measured for the saturating irradiance in thalli from the different Ci treatments, which increased significantly after the LC to HC shift and decreased nonsignificantly after the HC to LC shift. However, the photosynthetic efficiency α showed no significant alterations under different Ci conditions (Fig. 2). In all cases, new levels of different photosynthetic parameters were observed 24 h after the shifts, indicating a rather rapid acclimation of the light process to different Ci conditions in *C. braunii*, whereas pigmentation remained unchanged during the shift periods (Table S6).

**Fig. 3:**
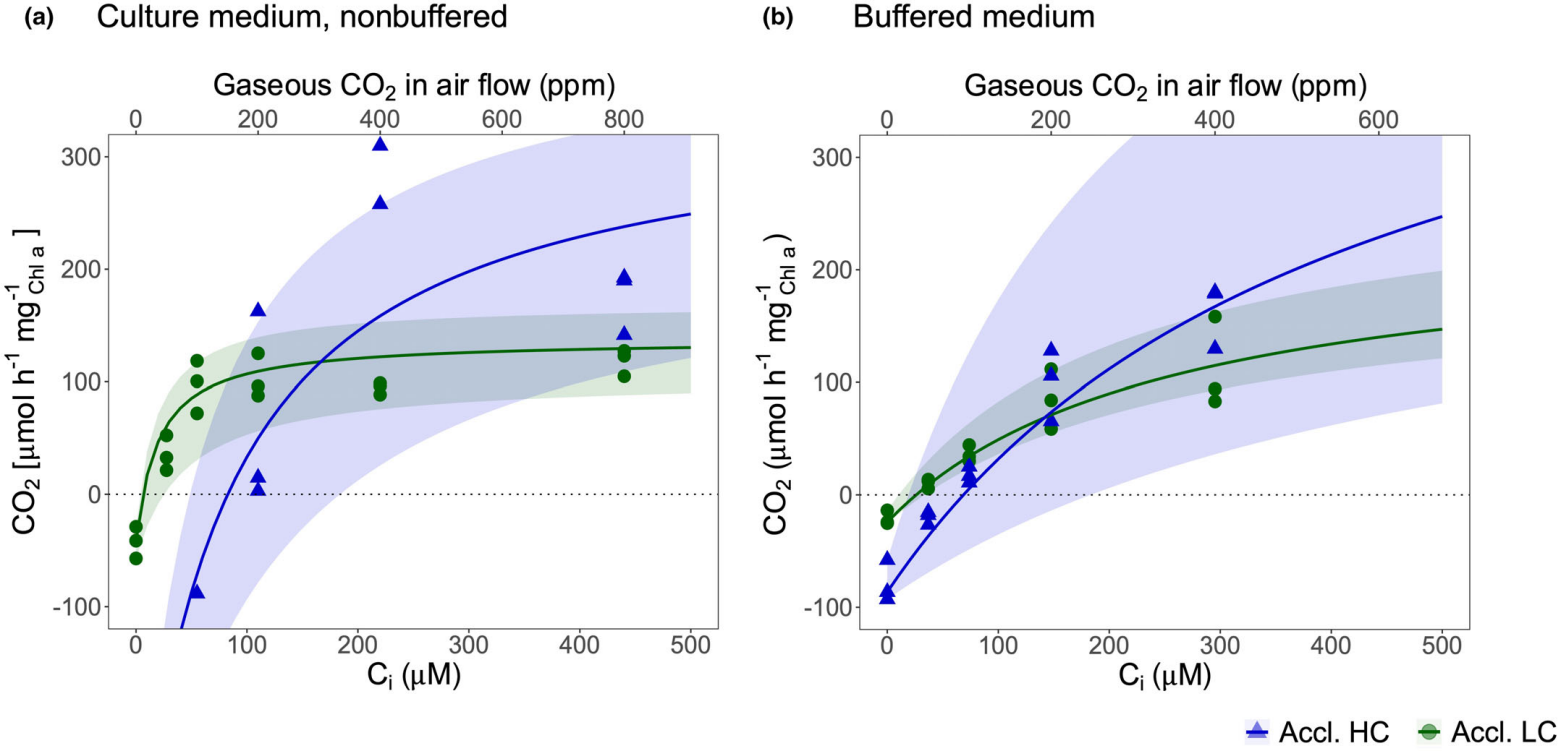
CO_2_ dependence of photosynthesis of *Chara braunii*. Photosynthetic CO_2_ uptake of low‐carbon‐acclimated (Accl. LC, green line and symbols) or high‐carbon‐acclimated (Accl. HC, blue line and symbols) *C. braunii*, measured with increasing CO_2_ levels (gaseous CO_2_ in air flow) at saturating light (1000 μmol photons m^−2^ s^−1^) and ambient O_2_ partial pressure (*n* = 3). (a) Measurements in ambient, nonbuffered medium. The thalli were directly incubated in the culture medium used for LC‐ or HC‐growth. (b) Instead of using cultivation medium, thalli were suspended in buffered medium (pH 7) during the measurements. Throughout the measurements, continuous airflow was given into the chamber stirring the media. The top axis represents gaseous CO_2_ in the airflow provided to the chamber given in ppm. The bottom axis represents total inorganic carbon (Ci) in μM. Solid lines represent fitted sample median as described, and shaded areas show whole sample variation. Symbols represent individual data points. The applied CO_2_ concentrations were converted to Ci concentrations as described in the Materials and Methods section.

In addition to the light reactions, the photosynthetic response to different Ci levels was characterized using a LI‐COR aquatic chamber that allows the direct measurement of algal CO_2_ fixation at different pCO_2_ levels under saturating light conditions. Interestingly, the CO_2_/photosynthesis curves were different for *C. braunii* thalli acclimated to either LC or HC conditions (Fig. 3a), resembling similar findings in CCM‐proven algae (Goudet *et al*., 2020). The curves indicated that thalli from the LC medium apparently had a higher CO_2_ affinity with a lower CO_2_ compensation point, while maximum photosynthesis was higher in HC‐acclimated *C. braunii* thalli. From these curves, the Km_CO2_ and the CO_2_ compensation point Γ were calculated. Indeed, we calculated that Γ was significantly lower in LC‐grown than in HC‐acclimated algae (Table 1), whereas the difference in CO_2_ affinity (*P* = 0.07) failed to pass our significance threshold. These data provided the first evidence that *C. braunii* uses a CCM under LC conditions, that is when grown in ambient air.

**Table 1:** Estimate of Ci‐dependent photosynthetic parameters in *Chara braunii.*

	Km (μM)	CO_2_ compensation point Γ (μmol l^−1^)	*V*_max_ (μmol h^−1^ mg^−1^ Chl*a*)	δ^13^C (‰)
Culture medium				
LC	28.5 ± 14.8	8.5 ± 3.8	178.9 ± 18.9	−17.3 ± 3.6
HC	82.2 ± 26.1	92.1 ± 28.7*	700 ± 72.5*	−30.0 ± 1.9*
Buffered medium				
LC	244.1 ± 25.9	21.7 ± 8.7	262 ± 40.5	
HC	390.4 ± 139.4	61.7 ± 14.0	583.5 ± 217.0*	

The table displays mean values ± SD (*n* = 3) of physiological parameters characterizing the photosynthetic Ci assimilation of *C. braunii* samples. Statistical differences were tested using the Shapiro–Wilk normality test and Levene's test for homogeneity of variance (center = median), followed by ANOVA (Type II tests) and Tukey's multiple comparisons of means (95% family‐wise confidence level) and are shown in Supporting Information Table S9. δ^13^C significant differences were tested using a Wilcoxon rank‐sum test. Statistical significances are shown comparing the low carbon (LC) and high carbon (HC) conditions of the respective cultivation condition either labeled as culture medium or buffered medium (statistical significant differences are marked by *, *P* ≤ 0.05).

The Ci species CO_2_, bicarbonate, and carbonate are present in varying proportions in aquatic media depending on the pH, which was different in the media between LC and HC cultivations, giving rise to different CO_2_/bicarbonate ratios (Table S8). Therefore, the measurements were repeated in media buffered to pH 7.0. The CO_2_/photosynthesis curves were less different between LC‐ and HC‐grown thalli when measured in the buffered medium (Fig. 3b). The Γ remained lower, and the Ci affinity in LC‐acclimated thalli appeared somewhat higher, but these differences were not statistically significantly different from HC‐grown thalli (Table 1). These changes indicated that in addition to CO_2_, *C. braunii* thalli from LC conditions can better utilize bicarbonate, which is more abundant in the nonbuffered medium, reaching pH values *c*. 9 (Table S8) compared with the medium buffered at pH 7.

In addition, we measured the photosynthetic activities of LC‐ vs HC‐acclimated thalli at ambient CO_2_ concentrations in the presence of decreased (3%) and increased (40%) O_2_ partial pressure, which are supposed to inhibit and stimulate photorespiratory rates of *C. braunii*, respectively. Some indications of a higher photosynthetic activity of thalli acclimated to LC than to HC were observed at ambient CO_2_ and O_2_ partial pressures, while the difference became smaller at 3% O_2_ and increased at 40% O_2_ (Fig. S2). However, due to large deviations in the single measurements, these nonsignificant changes do not clearly support the assumption that LC‐acclimated thalli of *C. braunii* expressing a CCM have lowered photorespiratory activity.

**Fig. 4:**
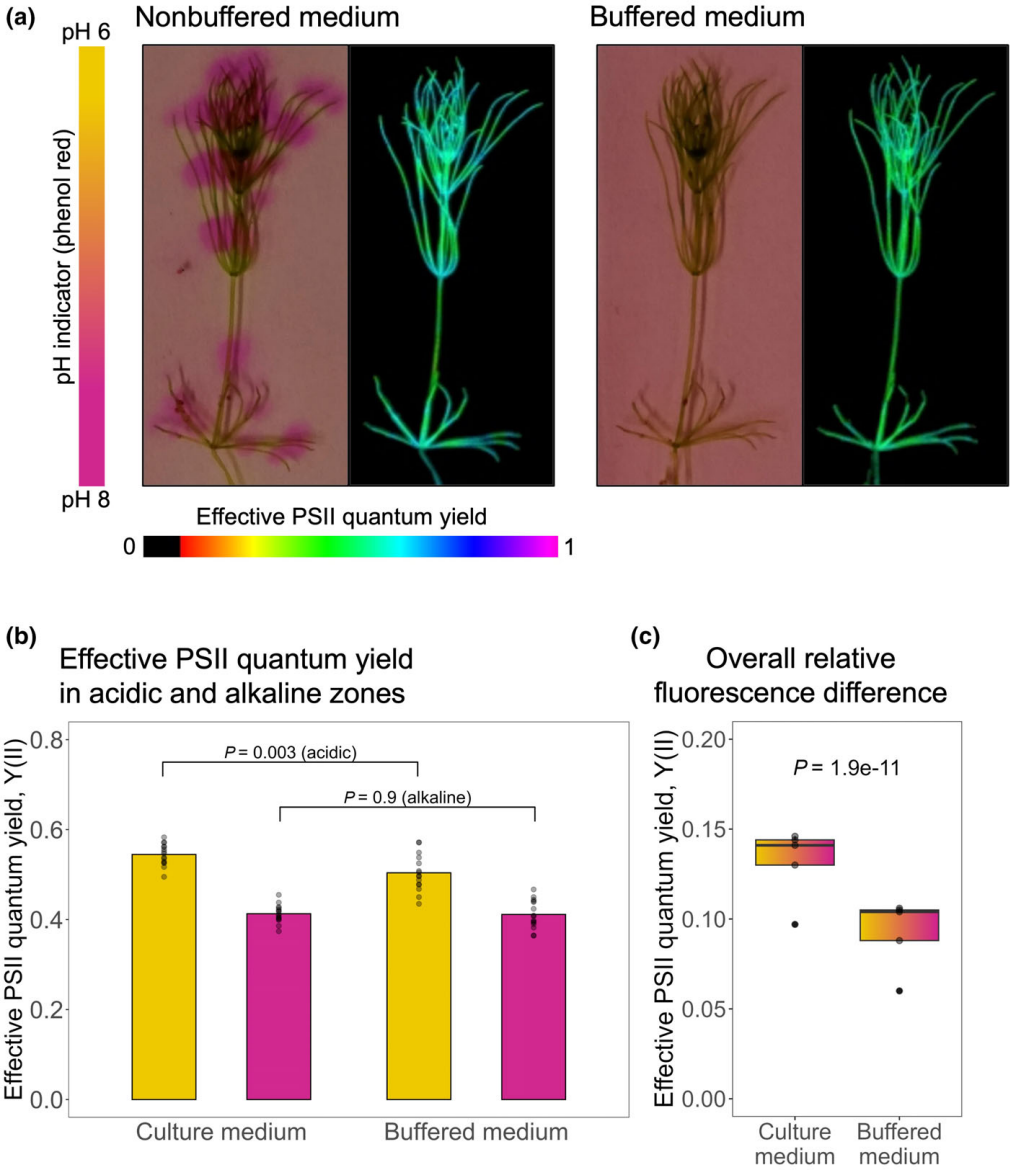
Chl fluorescence images reflecting the pH‐banding pattern in *Chara braunii*. (a) Images of *C. braunii* in nonbuffered culture medium colored with phenol red depicting acidic (yellow, pH ≤ 6) and alkaline (pink, pH ≥ 8) zones next to the respective pulse amplitude modulation (PAM) image and the same alga in buffered medium (pH 7) without a visible banding pattern and the respective PAM image. (b) Bar graphs of the mean effective photosystem II (PSII) quantum yield (YII) in the acidic (yellow) and alkaline (pink) zones of the respective medium, measured in three separate zones of five algal thalli. Individual data points are shown in gray. The *P*‐values show the comparison between the acidic band in culture and buffered medium (*P* = 0.003) and between the alkaline bands (*P* = 0.9) in the culture and buffered medium. (c) Overall difference of effective PSII quantum yield (YII) pooled within the respective medium shown as box plots stretching from the 25^th^ to 75^th^ percentile, black lines inside the boxes indicate the data median and whiskers represent 1.5× the interquartile range limits. Outliers are shown as black dots and individual data points are shown in gray. Statistical differences were obtained with a Welch two‐sample *t*‐test.

The characteristic banding of *Chara* spp. in acidic and alkaline zones has been suggested to be related to Ci acquisition. Therefore, we analyzed pH banding in *C. braunii* cultivated under ambient conditions at which LC‐acclimated thalli showed signs of a CCM. A clear zonation into acidic and alkaline parts of the thalli became visible when incubated with a pH indicator, which disappeared when the experiment was repeated in buffered medium (Fig. 4a). These thalli were analyzed in the ‘Imaging PAM’, which permits characterization of PSII activity in parts of intact macroalgae and plants. Zones with higher and lower PSII quantum yields appeared in the thallus of *C. braunii*, which remained less visible after transfer of the same thallus from the nonbuffered to buffered medium (Fig. 4a). Interestingly, the PSII quantum yield was significantly higher in acidic than in alkaline zones (Fig. 4c).

**Fig. 5:**
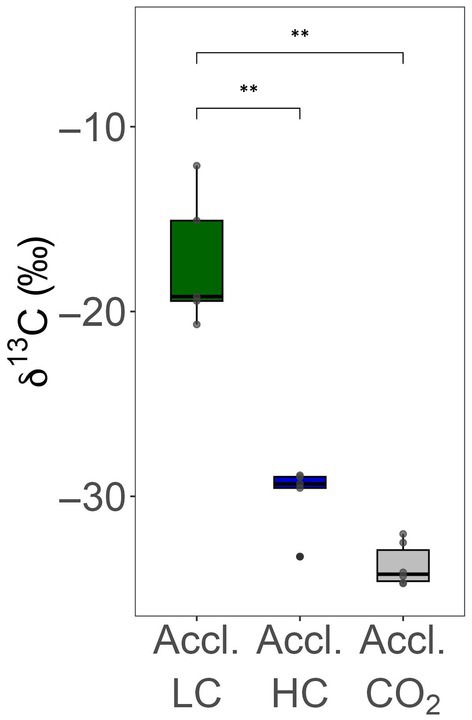
Carbon isotope discrimination in *Chara braunii* cultivated at different inorganic carbon (Ci) conditions. Algae were cultivated at ambient low‐Ci conditions (Accl. LC), in the presence of 10‐mM bicarbonate (Accl. HC), or with CO_2_ gassing (2% v/v, Accl. CO_2_). As described in the Materials and Methods section, isotope discrimination was related to the ^13^C values of the respective growth medium. Box plots of the isotopic composition of ^13^C from *C. braunii* biomasses range from the 25^th^ to 75^th^ percentile, black lines inside the boxes indicate the data median and whiskers represent 1.5× the interquartile range limits. Outliers are shown as black dots and individual data points are shown in gray. Statistical differences were tested by Shapiro–Wilk normality test followed by Wilcoxon rank‐sum test and are indicated with two asterisks, *P*‐value ≤ 0.01 (*n* ≥ 5). The measurements were taken in four biological replicates with two technical replicates each (*n* = 8). The dots indicate averages of the technical replicates.

Another indicator for the existence of the CCM is the δ^13^C level, as C3 plants lacking the CCM typically have δ^13^C levels of *c*. −30‰, similar to RubisCO, while an active CCM leads to higher, less negative δ^13^C levels (see Steensma *et al*., 2023, for reported δ^13^C levels). To investigate ^13^C discrimination, thalli of *C. braunii* were long‐term grown under LC or HC conditions, and the resulting biomass was analyzed. The biomass obtained under LC conditions had a δ^13^C level of *c*. −16‰, which is characteristic of photosynthetic organisms with a CCM. By contrast, when *C. braunii* was grown under HC in the presence of 10‐mM bicarbonate or with 2% CO_2_ gassing, the value dropped significantly to −30‰, which is indicative of organisms relying solely on RubisCO for CO_2_ fixation (Fig. 5). Collectively, the physiological characteristics demonstrated the presence of an active CCM in *C. braunii* when grown under ambient conditions, which was suppressed under HC.

### RNA‐seq characterization of Ci acclimation

To obtain a detailed view of Ci‐related gene expression changes, we performed an RNA‐seq experiment with *C. braunii* thalli under shifting Ci availability. The cDNA libraries were generated from total mRNA of the *C. braunii* samples in duplicates (Accl. LC, HC to LC after 48 h) and four triplicates. After sequencing, paired‐end read counts of 47 348 852 to 68 921 185 reads per sample were obtained. Following trimming and mapping steps, 5227 422 to 12 096 652 reads remained unmapped to the *Chara* genome, whereas 37 826 770 to 56 504 427 reads were uniquely mapped. Finally, genes with significantly altered expression were identified (Fig. S3).

**Fig. 6:**
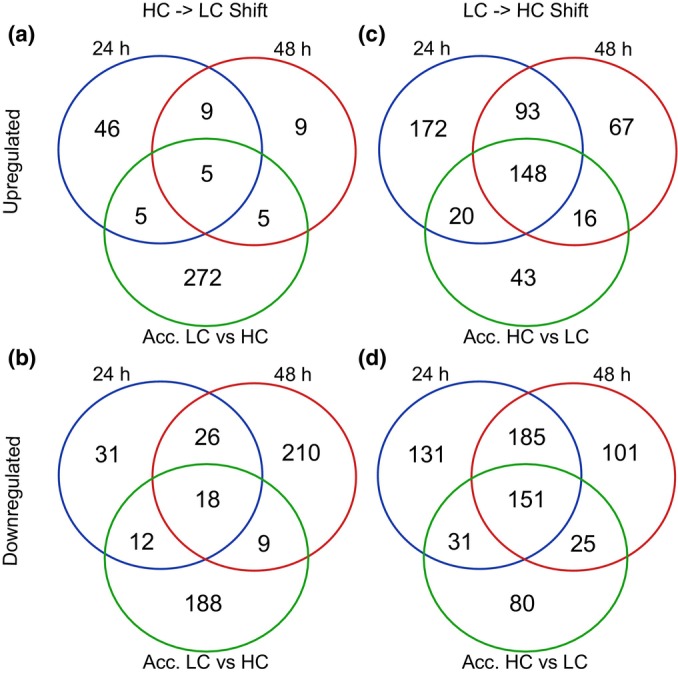
Differential gene expression in *Chara braunii* under different inorganic carbon (Ci) regimes. Left panel: Venn diagrams of numbers of (a) significantly up‐ or (b) downregulated genes after shift from high‐inorganic carbon (HC) to low‐inorganic carbon (LC) for 24, 48 h, or long‐term acclimation to LC vs HC. Right panel: Venn diagrams of numbers of (c) significantly up‐ or (d) downregulated genes after shift from LC to HC for 24, 48 h, or long‐term acclimation to high vs low Ci.

Mapped reads matched a median of 17 545 ± 481 putative genes. However, not all genes were transcribed at every time point; only a core of *c*. 14 290 genes was reproducibly found expressed in all samples (Fig. S4). Both the responses to changing Ci conditions for 24 and 48 h and the steady‐state comparisons in acclimated thalli were predominantly consistent between replicates (Fig. S5). Manual inspection of differentially expressed genes revealed that some belonged to retrotransposons or other repeated DNA elements. These genes were omitted from further analyses. The final list of differentially regulated genes (log_2_FC ≥ 1, *P‐*value ≤ 0.05) in *C. braunii* (Tables S1–S5) comprised 514 bicarbonate‐stimulated genes, among which 433 and 324 genes were elevated at 24 and 48 h after the addition of bicarbonate, respectively. A total of 498 and 462 genes were significantly downregulated at 24 and 48 h after the LC to HC shift, respectively (Fig. 6). Conversely, the expression of 65 and 28 genes was elevated, and of 87 and 263 genes downregulated at 24 and 48 h after the shift to LC, respectively. A subset of 241 genes was significantly upregulated at both HC time points, whereas 14 genes were significantly upregulated at both time points under LC. The comparison between thalli acclimated to permanent high‐ or low‐Ci availability revealed 287 genes with elevated and 227 with diminished expression at LC (Fig. 6).

**Fig. 7:**
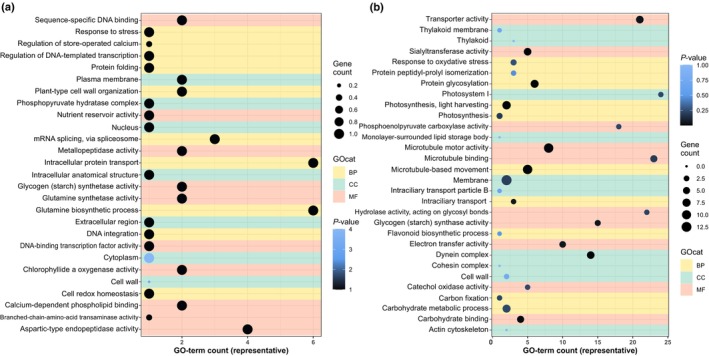
Gene Ontology (GO) term analysis of inorganic carbon (Ci)‐dependent gene expression changes in *Chara braunii* thalli. Dot plot representations of most overrepresented GO terms among differentially expressed genes after long‐term acclimation in high‐inorganic carbon (HC) vs low‐inorganic carbon (LC) (Accl. HC vs Accl. LC). GO terms were sorted by (a) up‐ and (b) downregulation and colored by category (GOcat); yellow for ‘biological process’, green for ‘cellular component’, and light red for ‘molecular function’. A semantic similarity threshold of 0.4 was applied to select GO term representatives. The *x*‐axis indicates the number of GO terms represented by the plotted identifiers, the dot size represents the number of differentially expressed genes associated with the GO term, and the color of the dots represents the adjusted *p*‐value per representative.

The genes identified as up‐ or downregulated at the different Ci regimes (Tables S1–S5) were used for GO term analysis to identify the physiological processes most important for Ci acclimation in *C. braunii*. Top terms of differentially expressed genes during the comparison of steady‐state HC‐ and LC‐cultivated thalli included protein phosphorylation/kinase activities, transmembrane transport/membrane, and several catalytic activities, particularly carbohydrate metabolism, ATP binding, and oxidoreductase activities (Fig. 7). The top terms of upregulated genes during the HC to LC shift by gene count were the biological processes transport, including metal ions, cell wall organization/modifications including pectin esterases, and several metabolic activities, particularly malate‐related (malate synthase, PEP carboxykinase) and gluconeogenesis (Fig. S6). By contrast, downregulated GO terms for HC‐ to LC‐shifted thalli were dominated by oxidoreductases, carbohydrate metabolism, and calcium‐ion binding (Fig. S7). Similar GO terms were dominant among the upregulated genes in thalli from LC‐ to HC‐shifted cells, which also contained genes for transmembrane transport and carbohydrate metabolism (Fig. S8). The downregulated genes after the LC to HC shifts were comprised of many genes encoding proteins involved in protein binding, several catalytic activities, and transmembrane transport (Fig. S9).

**Fig. 8:**
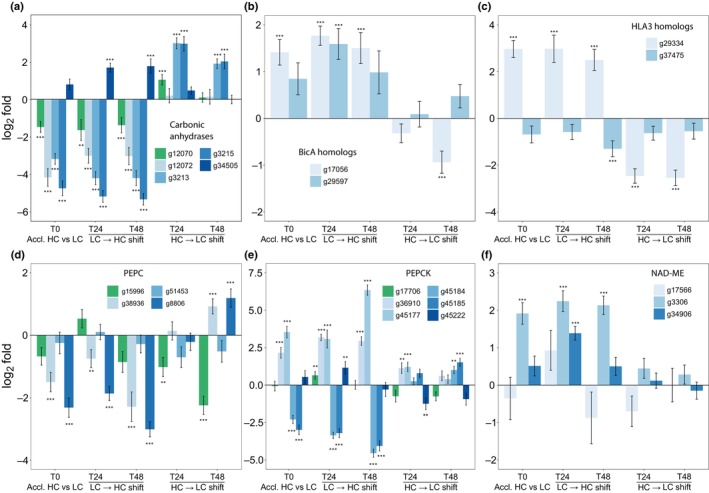
Expression of selected genes encoding proteins potentially involved in *Chlamydomonas*‐like or C4‐plant‐like CO_2_‐concentrating mechanisms in *Chara braunii* under different inorganic carbon (Ci) regimes. (a) Different carbonic anhydrases. (b) Putative Na^+^/bicarbonate symporters BicA. (c) Putative bicarbonate transporters HLA3. (d) Putative PEP carboxylases. (e) Putative PEP carboxykinases. (f) Putative NAD‐dependent malic enzymes. Mean values and SD are shown (*n* = 3). Statistically significant differences are marked (2 asterisks, *P*‐value ≤ 0.05; 3 asterisks, *P*‐value ≤ 0.0; Students *t*‐Test). HC, high carbon; LC, low‐carbon.

Our study focused on whether *C. braunii* performs a CCM. The above‐described physiological experiments provided clear evidence that thalli grown under ambient conditions use a CCM for efficient carbon fixation. Therefore, the transcriptome dataset was screened for genes encoding proteins that are known to be involved in the CCM of algae and/or C4 plants to gain insight into a possible underlying mechanism. Indeed, many Ci‐regulated genes are likely to encode CCM proteins (Table S10). A closer inspection of the Ci‐regulated genes showed that they responded with different kinetics toward the Ci regimes (Fig. 8).

Bicarbonate transporters such as HLA3 (hig‐light‐activated protein 3) or BicA (sodium‐dependent bicarbonate transporter A) and CAs play important roles in the biophysical CCM of *Chlamydomonas* and other algae (Wang *et al*., 2015; Matsuda *et al*., 2017). At least seven CA‐encoding genes exist in the *C. braunii* genome. Among them, the genes g3213 and g3215 encode two closely related CA9 proteins, which were highly induced when thalli were shifted from HC to LC and showed the opposite response after the LC to HC shift (Fig. 8a). A similar, but lower response was observed for the eukaryotic‐type CA g12070, whereas other CA genes g10864, g24327, and g12072 showed increased expression after transfer to LC, but did not significantly respond in LC to HC shifts. Finally, there was one CA‐encoding gene, g34505, with an inverse regulation, highly expressed after transfer to HC and less expressed after shift to LC (Fig. 8a). Furthermore, we noticed marked Ci‐related expression changes in genes encoding aquaporins or major intrinsic proteins, which can potentially act as channels for CO_2_ diffusion (Groszmann *et al*., 2017). Expression of the aquaporin genes g55164 and g19886 was decreased in thalli acclimated to HC conditions compared with LC, whereas expression of the major intrinsic protein genes g19238 and g19242 was increased under HC (Table S10).

Two genes in the *C. braunii* genome encode BicA homologs: g17056 and g29597. The expression of g17056 increased when shifted from LC to HC and decreased after the HC to LC shift. The second BicA gene, g29597, was also induced after bicarbonate addition, but did not respond to HC/LC shifts (Fig. 8b). Two genes, g29334 and g37475, encode proteins with considerable similarity to the *Chlamydomonas* HLA3 bicarbonate transporter. The gene g29334 showed inverse responses to changes in the Ci regime; that is, its expression increased after shift from LC to HC, indicating that it was induced by the added bicarbonate, whereas its expression was significantly decreased after the HC to LC shift, matching the regulation of the BicA gene g17056. The other HLA3 gene, g37475, showed decreased expression under all conditions (Fig. 8c).

Interestingly, some genes encoding the small subunit of RubisCO (RbcS) and RubisCO activase (Rca) were downregulated after shifts from LC to HC (Tables S1, S2). It has been reported that the streptophytic green alga *Mougeotiopsis* appears to compensate for the lack of pyrenoid‐based CCM by an extremely high expression of homologs of RbcS and Rca, whereas related algae with a pyrenoid showed much lower expression values (Hess *et al*., 2022). Therefore, we checked our dataset for the total expression level of the *rbcS* genes. In fact, the *rbcS*‐encoding gene g37370 showed the second highest expression of all genes in our dataset, whereas the expression of other *rbcS* genes (g46359, g52657, and g19696) belonged to the 1% most highly expressed genes.

Significant changes were also observed in the expression of several genes encoding enzymes potentially able to act in a C4‐plant‐like CCM. Two genes encoding PEP carboxylase, g8806 and g38936, showed decreased expression under HC vs LC and were induced 48 h after the shift from HC to LC (Fig. 8d). By contrast, the expression of the PEP carboxylase gene g15996 became lower when shifted from HC to LC. Among the decarboxylating PEP carboxykinases, two very similar genes (g45185 and g45184) showed clearly diminished expression under LC vs HC and became elevated under the opposite HC to LC shift (Fig. 8e). However, there are also two genes encoding PEP carboxykinases (g36910 and g45177), which showed much higher expression under high than low‐Ci conditions. Finally, among the several genes encoding NAD‐dependent malic enzymes, only g3306 for a mitochondrial isoform was clearly Ci‐responsive, because it showed much higher expression under HC than LC conditions (Fig. 8f).

## Discussion

Our study presents evidence that *C. braunii* operates a CCM when cultivated under ambient, low‐Ci conditions, as has recently been shown for the extremophile red alga *Cyanidioschyzon merolae* (Steensma *et al*., 2023) and before for many other algae (Raven *et al*., 2012) as well as seagrasses (Capó‐Bauçà *et al*., 2022). The first evidence for the existence of a CCM was provided by measurements of photosynthetic CO_2_ assimilation at different Ci levels. Here, LC‐acclimated thalli were characterized by a significantly lowered CO_2_ compensation point as in C4 compared with C3 plants. We also observed a nonsignificant trend toward a higher Ci affinity of 28 μM for LC‐grown thalli compared to 82 μM for HC‐acclimated thalli (Table 1). A higher Ci affinity has also been reported for intact thalli of *C. corallina* grown at low Ci compared with Ci‐enriched conditions (Brechignac & Lucas, 1987). Interestingly, the *C. braunii* whole thallus mean Ci affinity of 28 μM appears higher than the CO_2_ affinities of 42–44 μM measured for RubisCO from *Chara* sp. and *Nitella* sp. in protein extracts at saturated RuBP amounts and under O_2_‐free conditions (Yeoh *et al*., 1981) but falls within this range considering the variability of our data. Our photosynthetic CO_2_ fixation measurements were performed in the presence of ambient 21% O_2_, when O_2_ and CO_2_ compete for RubisCO activity and not under O_2_‐free conditions. Hence, we can assume a better CO_2_ affinity of *C. braunii* thalli when measured in the absence of oxygen.

Furthermore, we observed that adjustment of the pH to 7 during the photosynthetic CO_2_ assimilation measurements, which strongly shifts the equilibrium between the different Ci species from bicarbonate toward CO_2_ (Table S8), had a negative impact on the Ci affinity. These data imply that, in addition to CO_2_, a substantial amount of bicarbonate can be used by the CCM in *C. braunii*. Correspondingly, several putative bicarbonate transporters are encoded in the genome of this alga (will be discussed later). As shown before for the CCM‐bearing red alga *C. merolae* (Steensma *et al*., 2023), we also measured a nonsignificant impact of oxygen on the CO_2_ fixation rate in HC‐acclimated *Chara* thalli, whereas LC‐acclimated thalli are apparently less affected by increasing oxygen partial pressure. The existence of a CCM in LC‐acclimated *C. braunii* thalli was finally supported by δ^13^C measurements. As reported for C4 plants or microalgae with an active CCM, *C. braunii* biomass acclimated to LC conditions showed δ^13^C values of −14 to −18‰, whereas HC‐grown thalli, either from bicarbonate‐supplemented or CO_2_‐sparked media, were characterized by significantly lower δ^13^C values *c*. −30‰, similar to C3 plants without a CCM activity. It has been shown that δ^13^C values and RubisCO features vary in different organisms especially in aquatic media (for a detailed discussion, see Steensma *et al*., 2023). However, the large difference in the carbon isotope composition between HC‐ and LC‐grown thalli of *C. braunii* should be mostly related to the saturation of RubisCO with CO_2_ due to the proposed CCM. Furthermore, the observed difference in δ^13^C values between low‐CO₂‐grown thalli (LC) and high‐CO₂‐grown thalli(HC), ranging from 12‰ to 16‰, also supports the view that a substantial fraction of Ci assimilation is based on bicarbonate utilization. The observed difference is larger than expected based solely on Rayleigh distillation and differences in apparent affinity for external Ci. At equilibrium, bicarbonate is enriched in the heavier ^13^C isotope compared with dissolved CO₂, with a δ^13^C difference of *c*. 7‰ (Kendall & Caldwell, 1998). Therefore, increased reliance on HCO₃^−^ as a carbon source under low‐Ci conditions could lead to higher δ^13^C values in the thalli.

Collectively, the physiological characterizations clearly suggested that Charophyceae, at least *C. braunii*, express an active CCM under ambient conditions. However, the CCM mode should differ from that reported for chlorophytes, such as *Chlamydomonas*, because of the absence of pyrenoid structures in *Chara* chloroplasts (Pickett‐Heaps, 1975). To obtain insight into possible CCM, we performed an RNA‐seq experiment to identify Ci‐responsive genes in *C. braunii* thalli when shifted to CCM‐inducing and CCM‐repressing conditions, that is from HC to LC and from LC to HC conditions, respectively. Several hundred genes appeared to be Ci‐regulated. The entire dataset of Ci‐responsive genes (Tables S1–S5) was screened for genes encoding homologs of proteins that can potentially function in a CCM (Table S10). As expected, many of the genes upregulated during the HC to LC shift encode transport proteins and enzymes, particularly malate‐metabolism‐related (Fig. S6), which are involved in the biophysical as well as biochemical CCMs from algae and plants, respectively. It should be noted that gene expression changes do not necessarily equate to functionality unless more specific studies are performed; therefore, our discussion of selected expression changes should be taken with caution.

Bicarbonate transporters play a central role in the biophysical CCM of algae and cyanobacteria (for reviews, see Wang *et al*., 2015; Hagemann *et al*., 2021). The *C. braunii* genome encodes two proteins with sequence and structural similarities to HLA3 (Fig. S10), an ATP‐dependent bicarbonate transporter from *Chlamydomonas* highly upregulated at LC (Fang *et al*., 2012). One of these genes (g29334) that was clearly Ci‐regulated (Fig. 8c) clustered in a phylogenetic analysis together with HLA3 from *Chlamydomonas* and further putative bicarbonate transporters (Fig. S11). However, it seems unlikely that the protein G29334 is crucial for the CCM of LC‐grown *C. braunii* because its expression was opposite to CCM induction; that is, it was more highly expressed under HC than LC conditions. Similarly, the bicarbonate transporter BicA homolog, g1706, showed elevated expression in HC and lowered expression in LC (Table S10), whereas the second BicA‐encoding gene was less Ci‐responsive. BicA bicarbonate antiporters have been characterized in cyanobacteria, in which they use a Na^+^ gradient (Price *et al*., 2004). But it cannot be excluded that the *Chara* BicA‐like transporters might use the proton motive force. Hence, it can be concluded that HLA3‐ or BicA‐like transporters are likely involved in the bicarbonate uptake to fuel the internal Ci pool of *C. braunii*. However, according to their expression changes, they are rather induced by the addition of bicarbonate, which was used in our study to mimic HC conditions. The decreased expression of these genes under LC conditions seems to indicate that the bicarbonate transporters play no important role in the CCM, but a biochemical stimulation of their bicarbonate transport activity or the involvement of other bicarbonate transporters under ambient conditions with active CCM cannot be ruled out.

**Fig. 9:**
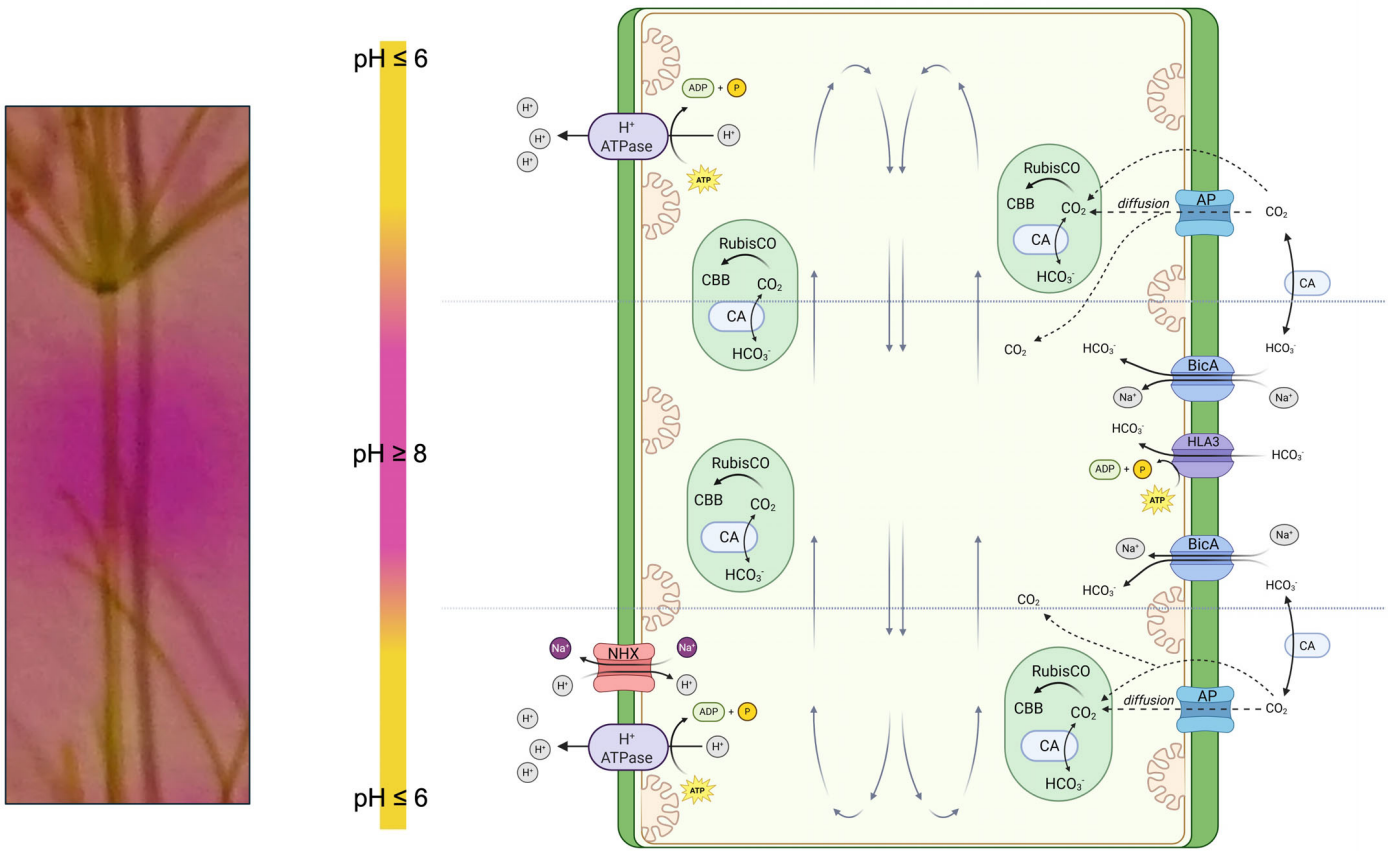
Tentative model of the CO_2_‐concentrating mechanisms in *Chara braunii*. The internodal cells of *C. braunii* show a banding in acidic and alkaline zones (left). At acidic zones, bicarbonate is converted into CO_2_, which then diffuses into the thallus via the membrane or aquaporin‐(AP)‐like channels. The fixation of CO_2_ by the Calvin–Benson–Bassham cycle sustains the diffusion gradient. At alkaline zones, bicarbonate is the dominating inorganic carbon (Ci) form, which can be transported into the thallus using the ATP‐dependent HLA3‐like transporter or the putative BicA‐like proton (sodium) symporter. Interconversion of bicarbonate and CO_2_ is enhanced by different carbonic anhydrases. Proton and sodium gradients are sustained by primary proton pumps (H^+^‐ATPase) and sodium/protein antiporters (NHX). Charasomes are indicated as invaginations of the plasma membrane supporting Ci uptake. The model was created using BioRender.com (https://BioRender.com/u27q827).

In addition to bicarbonate transporters, CAs that accelerate the pH‐dependent CO_2_/bicarbonate equilibrium play important roles in the CCM. Different types of CAs exist in algae and plants, which are involved not only in CCM but also in pH regulation (DiMario *et al*., 2018). In *C. braunii*, we identified three genes encoding CAs, g3213, g3215, and g12070, which showed clearly higher expression under LC than HC conditions. However, there was one, g34505, that showed the opposite behavior, that is a higher expression under HC than under LC (Fig. 8a). In *Chlamydomonas*, multiple CAs are involved in the CCM due to the need for rapid equilibration of Ci species in different cellular compartments (for a review, see Wang *et al*., 2015). Here, the LC‐induced CAs encoded by g3213, g3215, and g12070 were all predicted to be exported into the extracellular space, whereas the CA G34505 might be targeted into mitochondria or the thylakoid lumen. We conclude that *C. braunii* expresses a number of different Ci‐regulated CAs that can be found in different cellular compartments and are likely involved in the CCM of thalli cultivated under ambient conditions (Fig. 9). Furthermore, we observed marked Ci‐related changes in the expression of genes g55164 and g19886 encoding aquaporins, which showed lower expression in thalli acclimated to HC compared to LC. These aquaporins might facilitate Ci uptake because aquaporin‐like proteins can act as channels for CO_2_ diffusion (Groszmann *et al*., 2017). As channels, aquaporins can likely decrease the conductance of membranes for CO_2_, thereby accelerating the CO_2_ equilibrium between inside and outside. This will not increase the CO_2_ amount inside the cell due to the passive nature of the transport but can contribute to faster replenishment of internal CO_2_ fixed by RubisCO from the surrounding medium. Hence, it seems likely that extra‐ and intracellular CAs convert bicarbonate into CO_2_, which then diffuses into the thalli through the LC‐induced aquaporins (Fig. 9).

In addition to proteins potentially acting in a biophysical CCM as in *Chlamydomonas*, we identified many Ci‐responsive genes encoding proteins known to be important for the biochemical CCM of C4 plants (e.g. Pardo & VanBuren, 2021; Clapero *et al*., 2024). In this regard, two genes for the bicarbonate‐fixing PEP carboxylase, g8806 and g38936, were clearly induced under LC conditions and significantly lower expressed under HC. Furthermore, the decarboxylating PEP carboxykinases, encoded by g45185 and g45184, showed a similar Ci‐dependent pattern. Hints for a C4‐plant‐like CCM in some diatoms have been reported by Reinfelder *et al*. (2000). Recently, the contribution of PEP carboxylases in the CCM of *Phaeodactylum* has been analyzed using mutant strains, which indicated that a mitochondrial PEP carboxylase isoform seems to play some minor role in the CCM of this alga (Yu *et al*., 2022), while another study did not find indications for the direct involvement of this pathway in the diatom CCM (Haimovich‐Dayan *et al*., 2013). Thus, at first glance, the expression data might indicate that PEP carboxylase could contribute to the transient uptake and storage of Ci in the form of dicarboxylic acids within the thallus, while various PEP carboxykinases and NAD‐specific malic enzymes may be involved in the release of CO_2_ from dicarboxylic acids in the vicinity of RubisCO, thereby contributing to the CCM in *C. braunii*. However, a closer look makes this simple conclusion rather unlikely, because those PEP carboxylases that efficiently convert PEP with bicarbonate to oxaloacetate in the C4 plant metabolism carry two specific amino acids that make them highly processive and not inhibited by C4 acids (Paulus *et al*., 2013). A comparison of PEP carboxylases, including C4 metabolism‐specific enzymes, revealed that the *C. braunii* PEP carboxylases are similar to C3 plant enzymes, lacking the two C4‐specific amino acid substitutions (Fig. S12). Therefore, the Ci‐regulated enzymes involved in malate metabolism are likely not key components of the CCM but may contribute to some pH buffering to compensate for the altered cellular Ci composition. Similar conclusions were reported for *C. merolae*, in which also several enzymes known from the C4 plant metabolism were also Ci‐regulated, but probably not involved in the CCM (Steensma *et al*., 2023). Finally, the extremely high expression of RubisCO small subunit genes, especially under LC in *C. braunii*, may also contribute to compensate for the lack of pyrenoid‐based CCM, as has been reported previously for the streptophytic green alga *Mougeotiopsis* (Hess *et al*., 2022).

Our data, in combination with previous results regarding Ci uptake and assimilation among *Chara* spp., suggest an integrative model for the CCM in Charophyceae, particularly *C. braunii*, to efficiently utilize CO_2_ and bicarbonate under ambient conditions (Fig. 9). Generally, most authors agree that the above‐described pH‐banding pattern (Fig. 4) is a direct consequence of photosynthetic CO_2_ uptake (for reviews, see Beilby *et al*., 2022; Raven *et al*., 2014). In brief, proton extrusion by the proposed H^+^ antiporter or, more likely, P‐type ATPases that are localized in specific membrane segments results in acidification of surface areas at *Chara* internodal cells (Pertl‐Obermeyer *et al*., 2018). The acidic pH will locally raise the pCO_2_ by the dissolution of bicarbonate. As described in more detail in Raven *et al*. (2014), the decrease of one pH unit will increase the CO_2_/HCO_3_^−^ ratio by 10, and the uncatalyzed bicarbonate CO_2_ conversion is also 10‐fold faster. Hence, the increased CO_2_ partial pressure at acidic zones promotes CO_2_ influx in the cell and provides a concentration of CO_2_ at the site of RubisCO. This results in better carboxylation activity in steady‐state photosynthesis, higher than in bulk medium, thereby acting as CCM. Internal and external CAs might accelerate this effect in addition. The passive CO_2_ diffusion through the cell membrane can be supported by increased expression of aquaporins, facilitating CO_2_ membrane passage (Groszmann *et al*., 2017). Inside the internodal cell, CO_2_ is trapped by the more alkaline pH, as has been proposed for the CCM in the acidophilic alga *C. merolae* (Steensma *et al*., 2023). At least under more basic conditions in the alkaline zones, bicarbonate transporters and other Ci pumps (as has been proposed in *C. tomentosa* by Ray *et al*., 2003) might also be involved and accumulate Ci inside the cell. Hence, the accelerated CO_2_ inward diffusion in acidic zones and the likely bicarbonate uptake into internodal cells then meet the photosynthetic demand of RubisCO in the chloroplasts. In giant *Chara* cells, the characteristic protoplasm movement can traverse acidic and alkaline regions, traveling along the cell wall and thus transporting the accumulated Ci along the cell membrane‐associated chloroplasts (Fig. S13). Finally, chloroplast CAs may help to convert accumulated bicarbonate into CO_2_. A direct effect of alkaline and acidic zones on PSII quantum yield, supporting this hypothesis, was demonstrated by Bulychev *et al*. (2001, 2003) and was also found in the present study (Fig. 4). Further studies are necessary to evaluate the contribution of other Ci‐regulated genes toward the acclimation of *C. braunii* to fluctuating Ci conditions.

## Competing interest

None declared.

## Author contributions

MH, HS and WRH conceptualized the study. CMH performed the cultivations and sampling with *C. braunii*, measured photosynthesis and evaluated data. PW and CMH measured inorganic carbon‐dependent photosynthesis and evaluated data. HS and MH supervised the evaluation of these experiments. CMH and MV performed delta ^13^C analyses and data evaluation. DAH generated RNA samples and performed all bioinformatic analyses. WRH supervised the evaluation of the bioinformatics data. MH and WRH drafted the manuscript, and all authors were involved in writing the final manuscript. CMH and DAH contributed equally to this work.

## Disclaimer

The New Phytologist Foundation remains neutral with regard to jurisdictional claims in maps and in any institutional affiliations.

## Supporting information


**Fig. S1** Experimental determination of CO_2_ solubility in the different liquid media.
**Fig. S2** Photosynthetic inorganic carbon uptake under different O_2_ concentrations.
**Fig. S3** Workflow of RNA‐Seq analysis.
**Fig. S4** Venn diagrams of raw gene reads.
**Fig. S5** Heatmap analysis of differential gene expression.
**Fig. S6** Gene Ontology term analysis of gene expression, upregulated after HC‐LC shift.
**Fig. S7** Gene Ontology term analysis of gene expression, downregulated after HC‐LC shift.
**Fig. S8** Gene Ontology term analysis of gene expression, upregulated after LC‐HC shift.
**Fig. S9** Gene Ontology term analysis of gene expression, downregulated after LC‐HC shift.
**Fig. S10** Domain structure and alignment of HLA3 homolog.
**Fig. S11** Phylogenetic analysis of the putative *Chara braunii* HLA3 homolog.
**Fig. S12** Partial sequence alignment of *Chara braunii* putative PEP carboxylases and plant enzymes.
**Fig. S13** Stationary chloroplasts in internodal cells of *Chara braunii*.

**Table S1** List of significantly up‐ or downregulated genes at time point 24 h after low‐to‐high CO_2_ shift (T24_LH).
**Table S2** List of significantly up‐ or downregulated genes at time point 48 h after low‐to‐high CO_2_ shift (T48_LH).
**Table S3** List of significantly up‐ or downregulated genes at time point 24 h after high‐to‐low CO_2_ shift (T24_HL).
**Table S4** List of significantly up‐ or downregulated genes at time point 48 h after high‐to‐low CO_2_ shift (T48_HL).
**Table S5** List of significantly up‐ or downregulated genes comparing samples from thalli acclimated to either high‐inorganic carbon or low‐inorganic carbonconditions (Accl_HC‐LC).
**Table S6** Pigmentation of *Chara braunii* used for the photosynthetic measurements at different inorganic carbon.
**Table S7** Calculated statistics of dual‐pulse amplitude modulation measurements displayed in Fig. 2.
**Table S8** pH values of media at different inorganic carbon values.
**Table S9** Calculated statistics of aquatic chamber measurements displayed in Table 1.
**Table S10** Summary of regulated gene numbers and expression of selected genes for putative CO_2_‐concentrating mechanism components.
Please note: Wiley is not responsible for the content or functionality of any Supporting Information supplied by the authors. Any queries (other than missing material) should be directed to the *New Phytologist* Central Office.

## Data Availability

The Illumina RNA‐seq data produced in this study have been deposited in the Sequence Read Archive under BioProject: PRJNA965918 (SRR27870321–SRR27870336) at https://www.ncbi.nlm.nih.gov/bioproject/PRJNA965918.
