# Supplementary material for: Evidence for a CO2 ‐concentrating mechanism in the model streptophyte green alga Chara braunii
Source: New Phytol. 2025 Jun 13;247(3):1218–33. doi: 10.1111/nph.70283 (PMC12222935; doi:10.1111/nph.70283)
Supplement: Supplementary file 1 — Fig. S1 Experimental determination of CO2 solubility in the different liquid media. Fig. S2 Photosynthetic inorganic carbon uptake under different O2 concentrations. Fig. S3 Workflow of RNA‐Seq analysis. Fig. S4 Venn diagrams of raw gene reads. Fig. S5 Heatmap analysis of differential gene expression. Fig. S6 Gene Ontology term analysis of gene expression, upregulated after HC‐LC shift. Fig. S7 Gene Ontology term analysis of gene expression, downregulated after HC‐LC shift. Fig. S8 Gene Ontology term analysis of gene expression, upregulated after LC‐HC shift. Fig. S9 Gene Ontology term analysis of gene expression, downregulated after LC‐HC shift. Fig. S10 Domain structure and alignment of HLA3 homolog. Fig. S11 Phylogenetic analysis of the putative Chara braunii HLA3 homolog. Fig. S12 Partial sequence alignment of Chara braunii putative PEP carboxylases and plant enzymes. Fig. S13 Stationary chloroplasts in internodal cells of Chara braunii. [file NPH-247-1218-s001.pdf]

***New Phytologist* Supporting Information**

Article title: **Evidence for a CO_2-concentrating mechanism in the model streptophyte green alga *Chara braunii***

Authors: Carolin M. Heise, Daniel Heß, Peter Walke, Maren Voß, Hendrik Schubert, Wolfgang R. Hess, Martin Hagemann

Article acceptance date: 15 May 2025

The following Supporting Information is available for this article:

Fig. S1 Experimental determination of CO_2 solubility in the different liquid media.

Fig. S2 Photosynthetic Ci uptake under different O_2 concentrations.

Fig. S3 Workflow of RNA-Seq analysis.

Fig. S4 Venn diagrams of raw gene reads.

Fig. S5 Heatmap analysis of differential gene expression.

Fig. S6 GO term analysis of gene expression, up-regulated after HC-LC shift.

Fig. S7 GO term analysis of gene expression, down-regulated after HC-LC shift.

Fig. S8 GO term analysis of gene expression, up-regulated after LC-HC shift.

Fig. S9 GO term analysis of gene expression, down-regulated after LC-HC shift.

Fig. S10 Domain structure and alignment of HLA3 homolog.

Fig. S11 Phylogenetic analysis of the putative *C. braunii* HLA3 homolog.

Fig. S12 Partial sequence alignment of *C. braunii* PEPC and plant enzymes

Fig. S13 Stationary chloroplasts in internodal cells of *Chara braunii*.

Supplementary Tables S1-S10 can be found in the separate Excel file (Supplementary Tables.xlc)

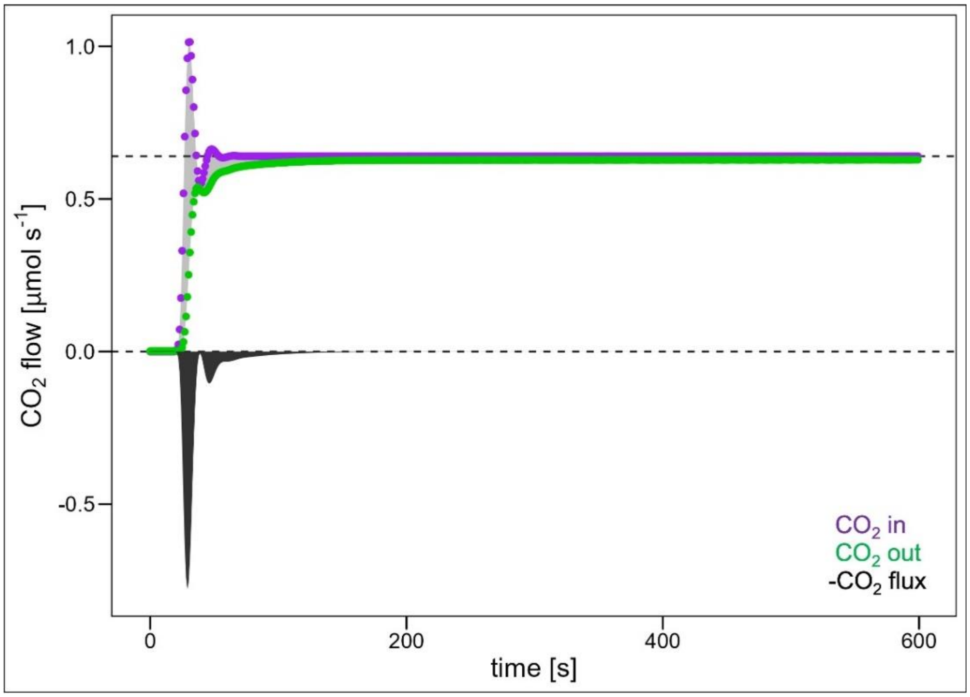

Fig. S1 Graphical representation of the experimental determination of CO_2 solubility in the different liquid media in the set up (LI-6800 aquatic chamber). First, the airflow bubbling through the medium was completely CO_2-depleted. Adjusting the inflow to defined concentrations (in the graph 1600 ppm) allows to observe the CO_2 uptake of the medium (depicted negative for better visibility, the integral represents the dissolved inorganic carbon Ci = DIC).

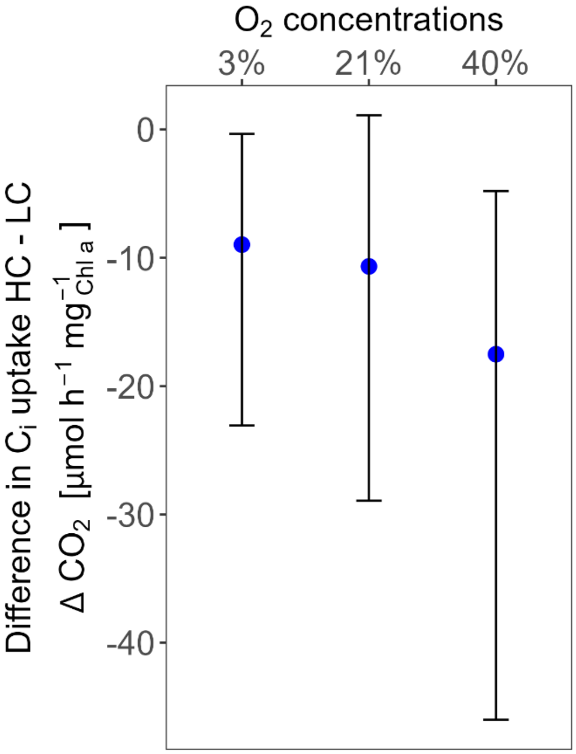

Fig. S2 Photosynthetic Ci uptake under different O_2 concentrations of *Chara braunii*. Photosynthetic CO_2 uptake was measured while supplying 3%, 21% and 40% O_2 (gaseous O_2 in air flow) at saturating light (1000 µmol photons m^{-2} s^{-1}) in the presence of continuous CO_2 (400 ppm). Measurements were performed with low carbon (LC) and high carbon (HC) acclimated algae. The blue dots show the mean values of HC (3 % = 20.80, 21 % = 21.70, 40 % = 22.61) subtracted by mean LC values (3 % = 29.77, 21 % = 32.37, 40 % = 40.13) for the respective oxygen concentrations (n = 3). The error bars indicate the maximum range of computable differences (HC_{max} – LC_{min}, HC_{min} – LC_{max}), while the blue dot represents the mean value.

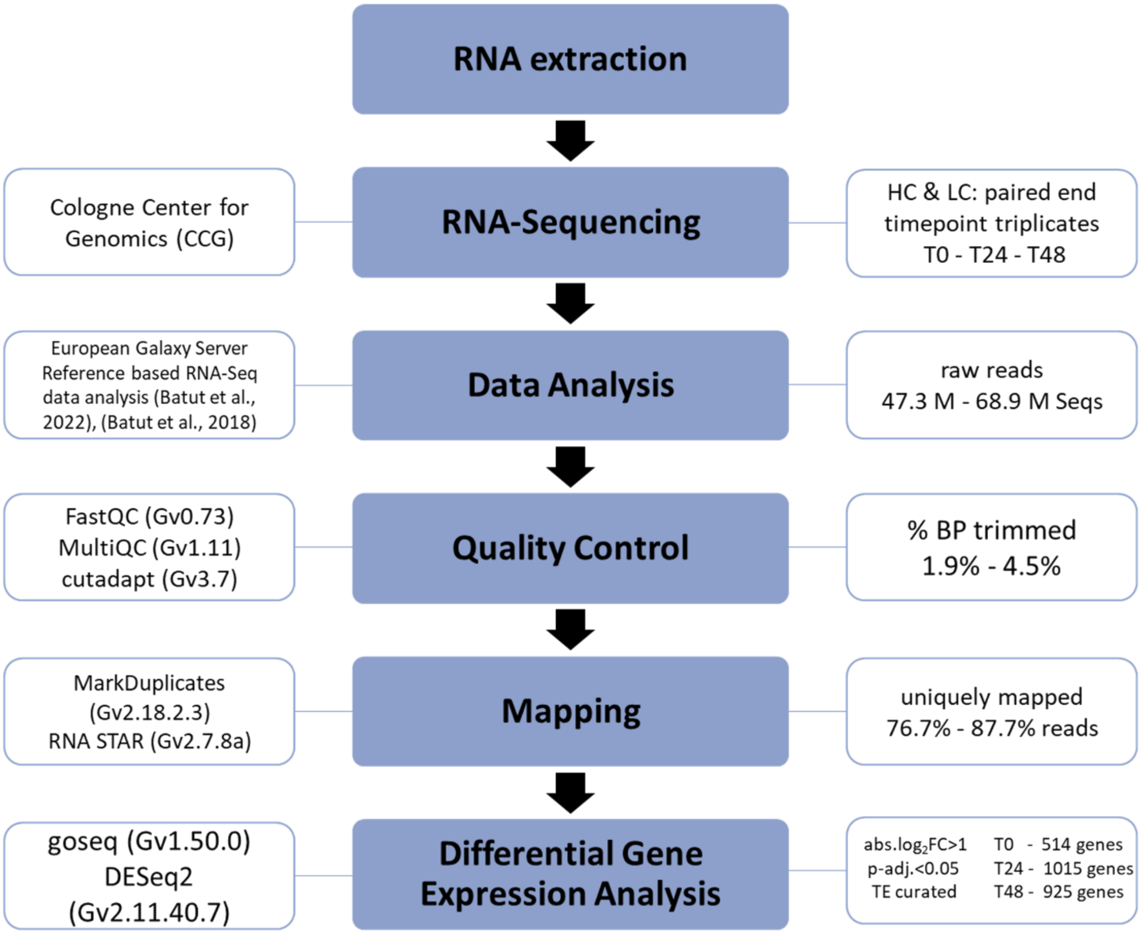

Fig. S3 Workflow of RNA-Seq analysis. Overview of the *Chara braunii* RNA-seq analysis methodology. Blue boxes (middle) illustrate the workflow process from RNA extraction to differential gene expression analysis, bordered boxes (left) illustrate the bioinformatic tools and utilized methods, bordered boxes (right) showcase the number of collected samples, raw, trimmed and mapped reads after every bioinformatic step. Analysis of data was performed on the European galaxy server (The Galaxy Community, 2022), based on the published guidelines for reference-based RNA-seq data analysis (Batut *et al*., 2018, 2022).

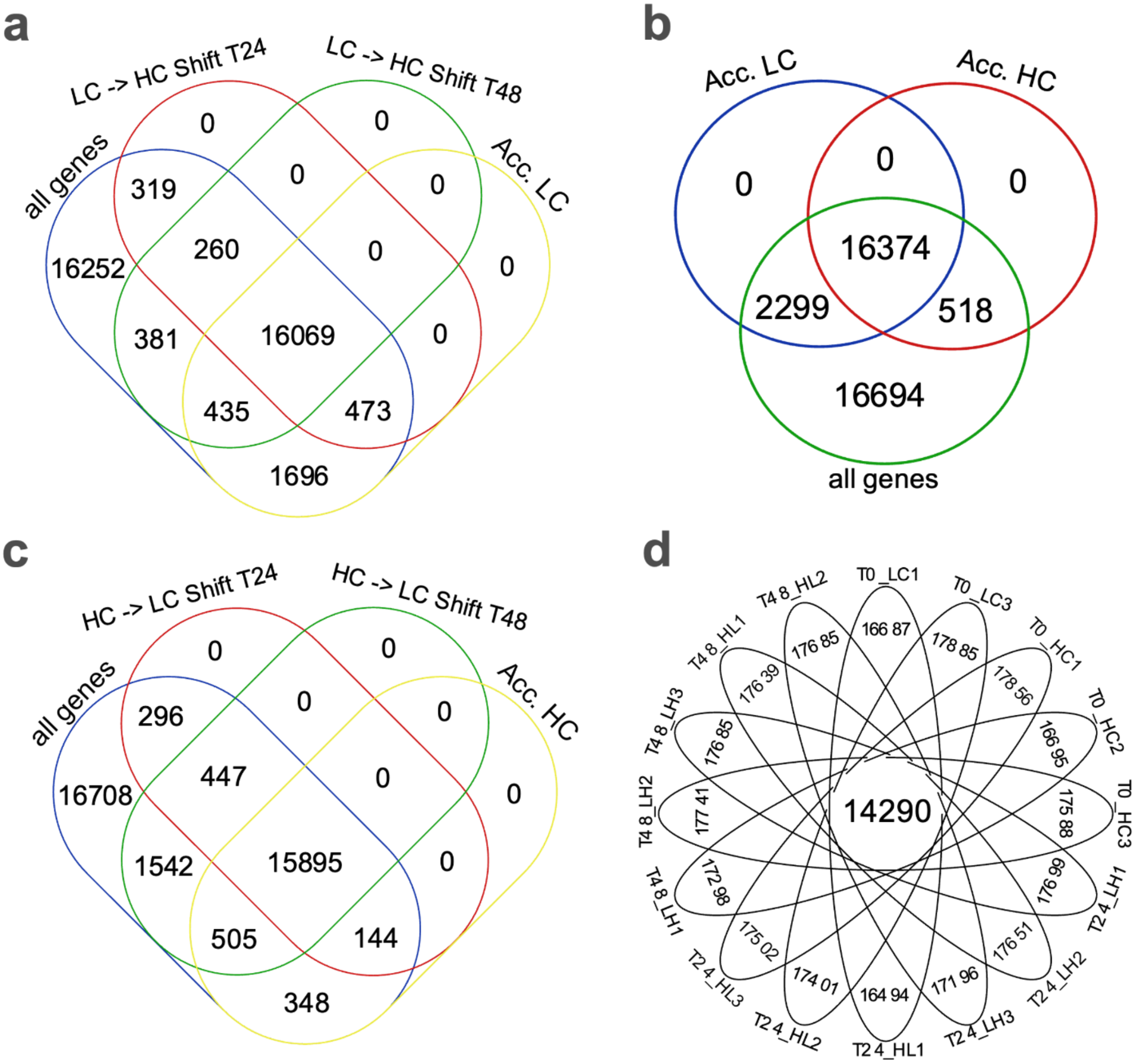

Fig. S4 Venn diagrams of raw gene reads. Venn diagrams of non-zero gene reads **(a. to c.** non-zero median reads per category), detected in the mapped transcriptome samples (RNA STAR). **d.** Raw non-zero gene reads per sample, as well as the number of expressed non-zero core genes found in every sample. The time points after HC to LC or LC to HC shifts are abbreviated as T0 for long-term acclimated samples, T24 and T48 for the shift after 24 and 48 h, respectively. HL indicates a shift from high to low and LH a shift from low to high carbon, with replicate numbers appended.

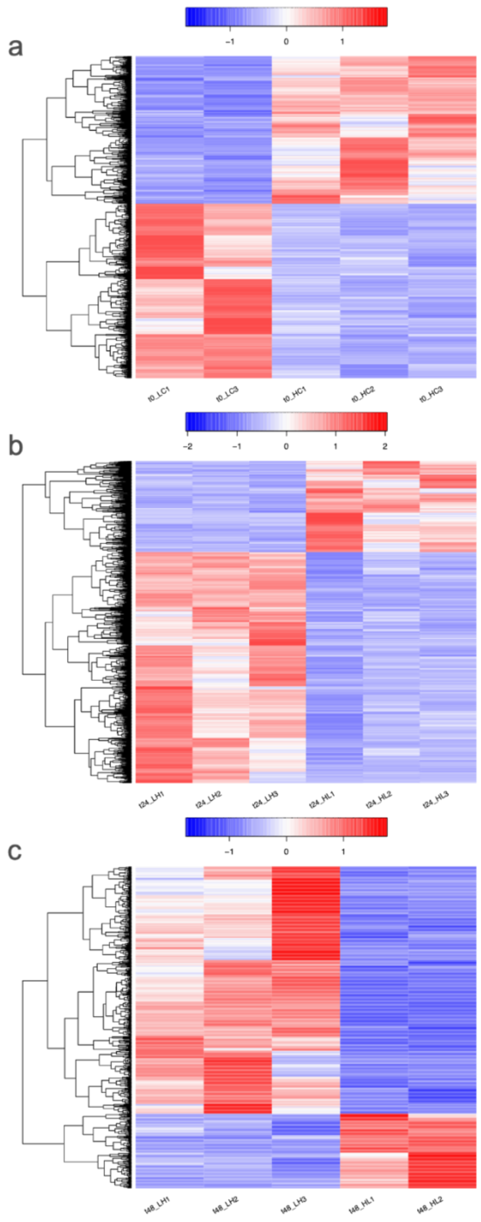

Fig. S5 Heatmap analysis of differential gene expression. Heatmaps showing genes differentially expressed (abs. log2FC>1, adj. p value<0.05) during at least one of the measured time points. Calculated Z-scores for mean normalized counts (blue to red) plotted for each sample. Dataset of shown genes was not curated regarding annotated transposable elements. **a.** Steady state comparison between acclimation to high (HC) and low (LC) inorganic carbon containing medium. **b.** Comparison between shifting Ci availability (high to low, HL, and conversely LH) after 24 and **c.** 48 hours.

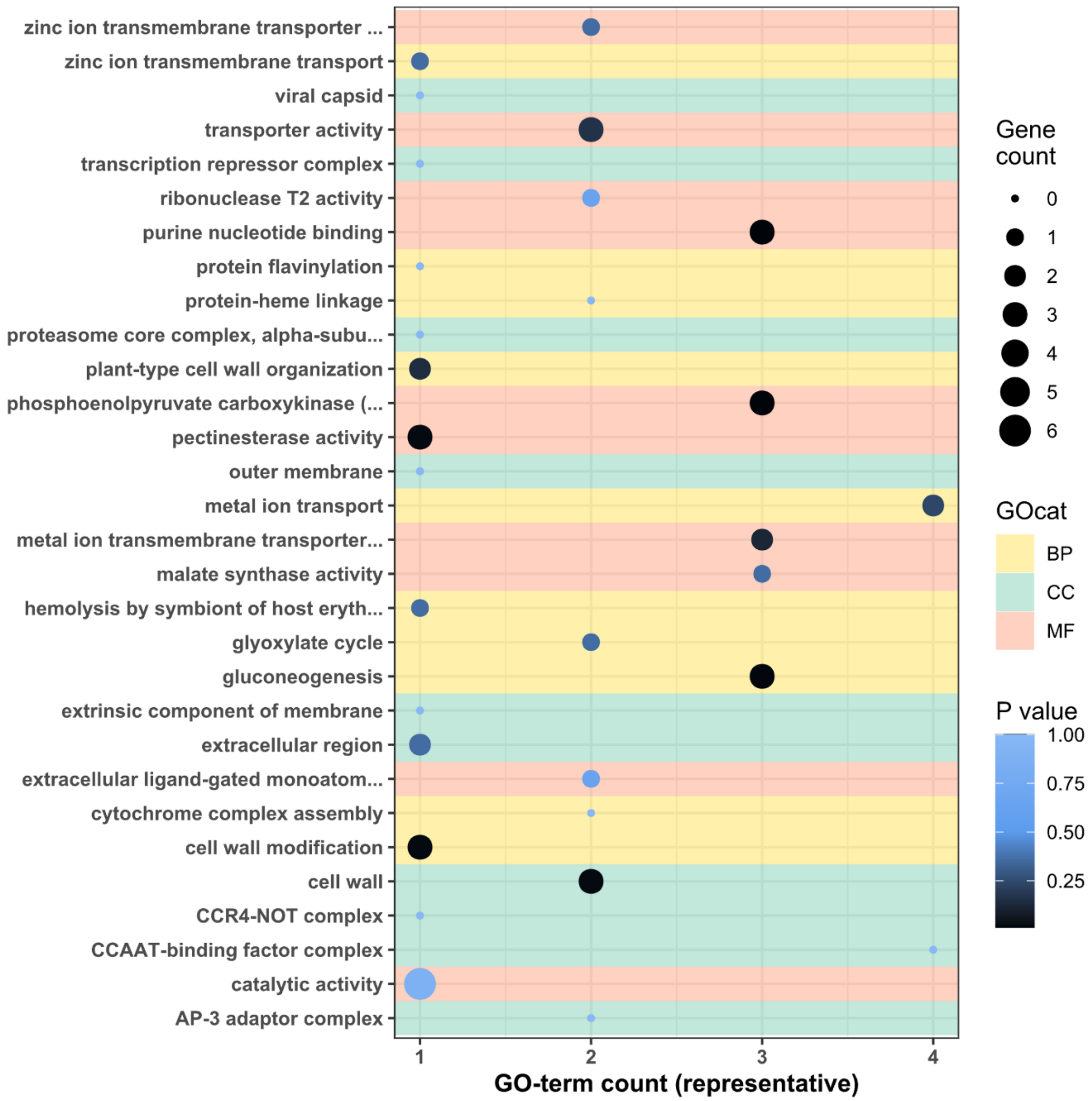

Fig. S6 GO term analysis of Ci-dependent gene expression changes in *Chara braunii* thalli. Dot plot representations of top overrepresented gene ontology terms enriched within up-regulated genes in *C. braunii* after HC to LC shifts for 24 and 48 h. Gene ontology terms were sorted by regulation and colored by category (GOcat); yellow for "biological process", green for "cellular component" and light-red for "molecular function". Semantic similarity thresholds were used to select GO term representatives (GO-Figure v.1.0.1); 0.8 was used for GO terms. The Y-axis (GO-term count (representative)) indicates the amount of GO terms represented by the plotted identifiers, the dot size represents the number of differentially expressed genes associated with the GO term, and the color of the dots represents the adjusted *p*-value per representative.

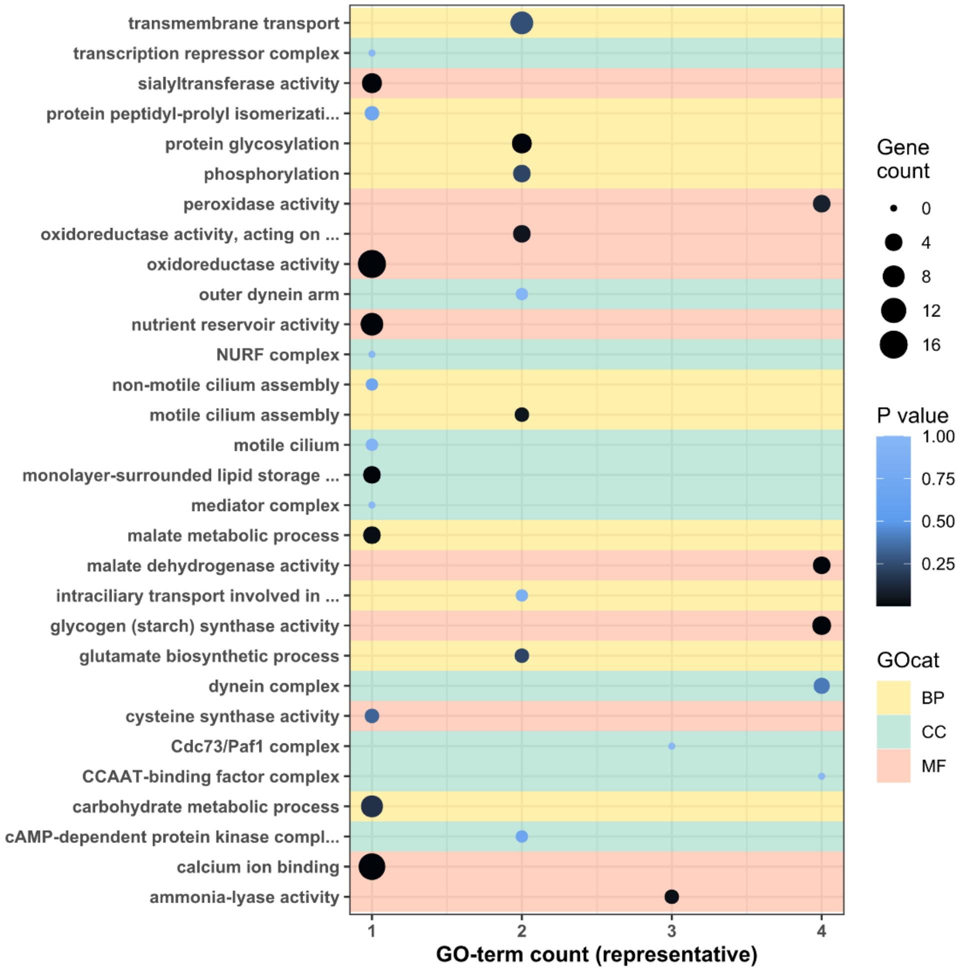

Fig. S7 GO term analysis of Ci-dependent gene expression changes in *Chara braunii* thalli. Dot plot representations of top overrepresented gene ontology terms enriched within down-regulated genes in *C. braunii* after HC to LC shifts for 24 and 48 h. Gene ontology terms were sorted by regulation and colored by category (GOcat); yellow for "biological process", green for "cellular component" and light-red for "molecular function". Semantic similarity thresholds were used to select GO term representatives (GO-Figure v.1.0.1); 0.8 was used for GO terms. The Y-axis (GO-term count (representative)) indicates the amount of GO terms represented by the plotted identifiers, the dot size represents the number of differentially expressed genes associated with the GO term, and the color of the dots represents the adjusted *p*-value per representative.

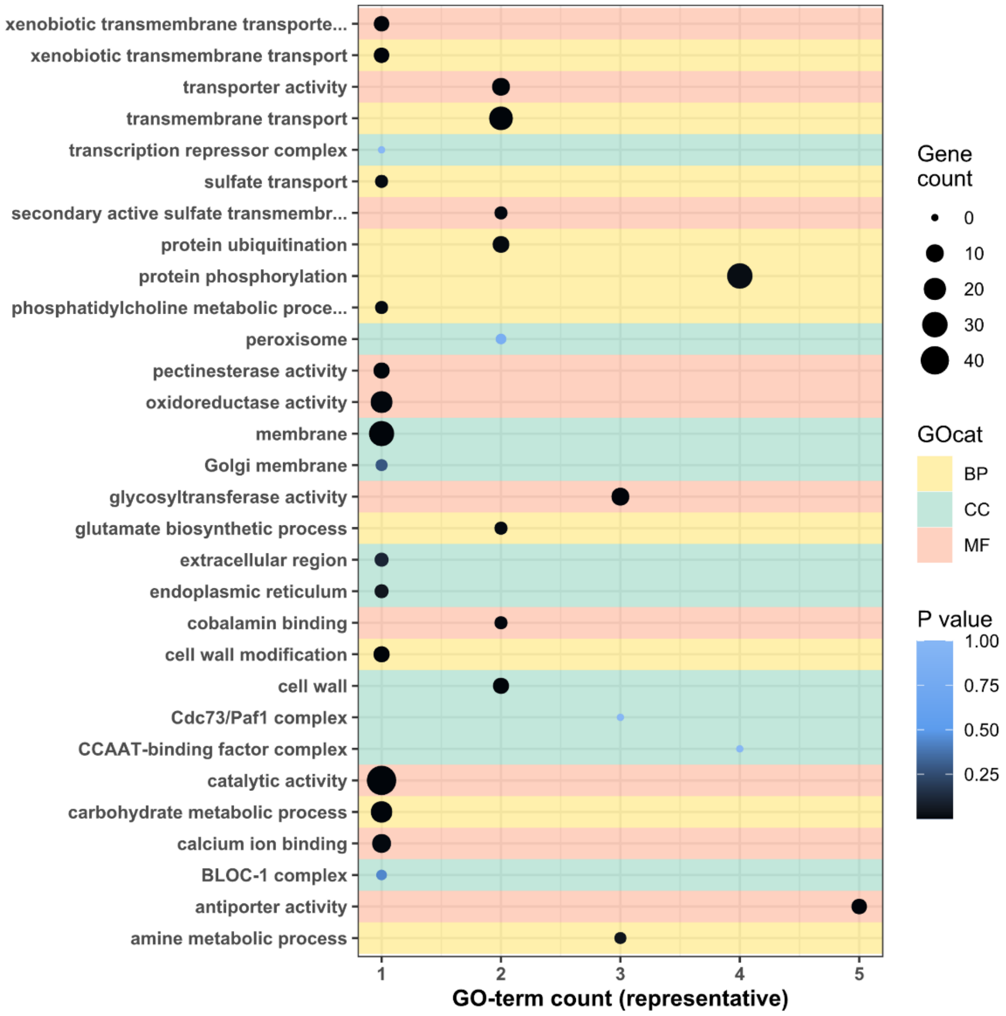

Fig. S8 GO term analysis of Ci-dependent gene expression changes in *Chara braunii* thalli. Dot plot representations of top overrepresented gene ontology terms enriched within up-regulated genes in *C. braunii* after LC to HC shifts for 24 and 48 h. Gene ontology terms were sorted by regulation and colored by category (GOcat); yellow for "biological process", green for "cellular component" and light-red for "molecular function". Semantic similarity thresholds were used to select GO term representatives (GO-Figure v.1.0.1); 0.8 was used for GO terms. The Y-axis (GO-term count (representative)) indicates the amount of GO terms represented by the plotted identifiers, the dot size represents the number of differentially expressed genes associated with the GO term, and the color of the dots represents the adjusted *p*-value per representative.

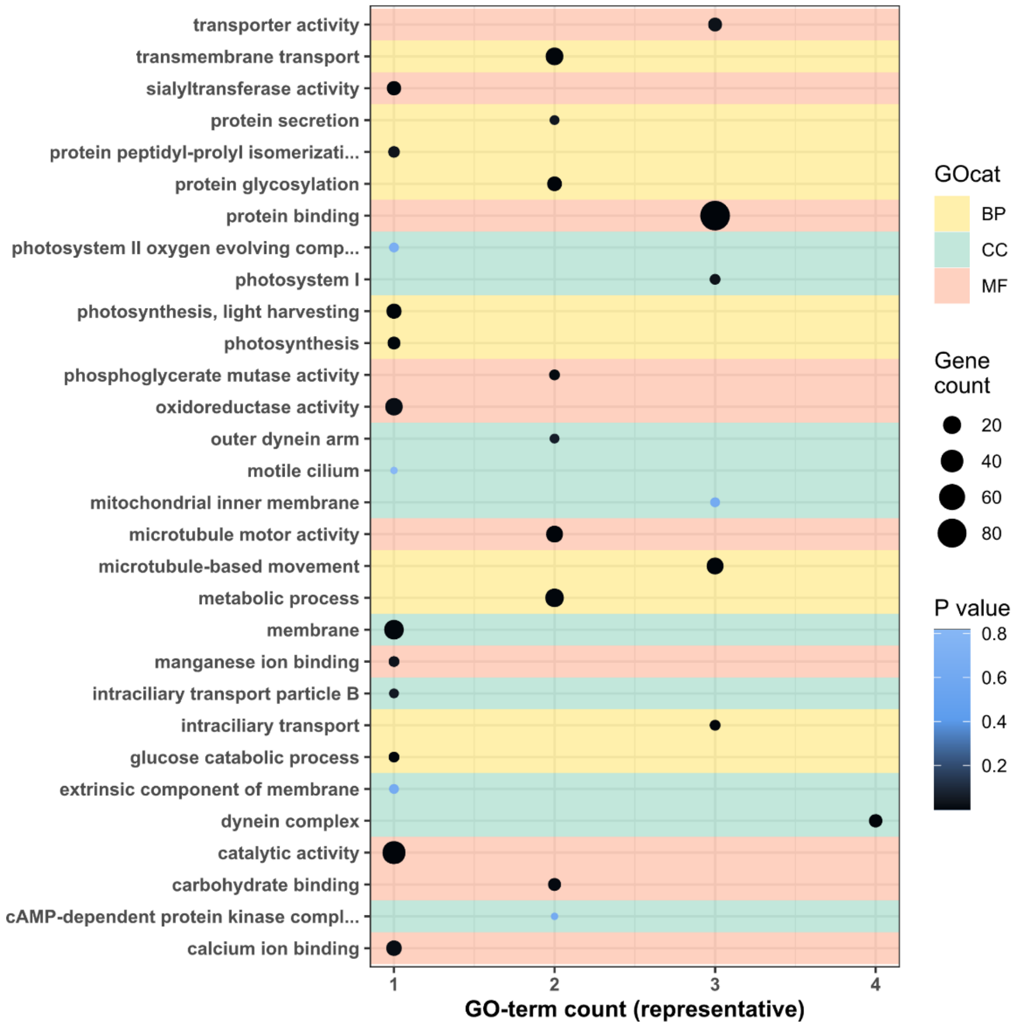

Fig. S9 GO term analysis of Ci-dependent gene expression changes in *Chara braunii* thalli. Dot plot representations of top overrepresented gene ontology terms enriched within down-regulated genes in *C. braunii* after LC to HC shifts for 24 and 48 h. Gene ontology terms were sorted by regulation and colored by category (GOcat); yellow for “biological process”, green for “cellular component” and light-red for “molecular function”. Semantic similarity thresholds were used to select GO term representatives (GO-Figure v.1.0.1); 0.8 was used for GO terms. The Y-axis (GO-term count (representative)) indicates the amount of GO terms represented by the plotted identifiers, the dot size represents the number of differentially expressed genes associated with the GO term, and the color of the dots represents the adjusted *p*-value per representative.

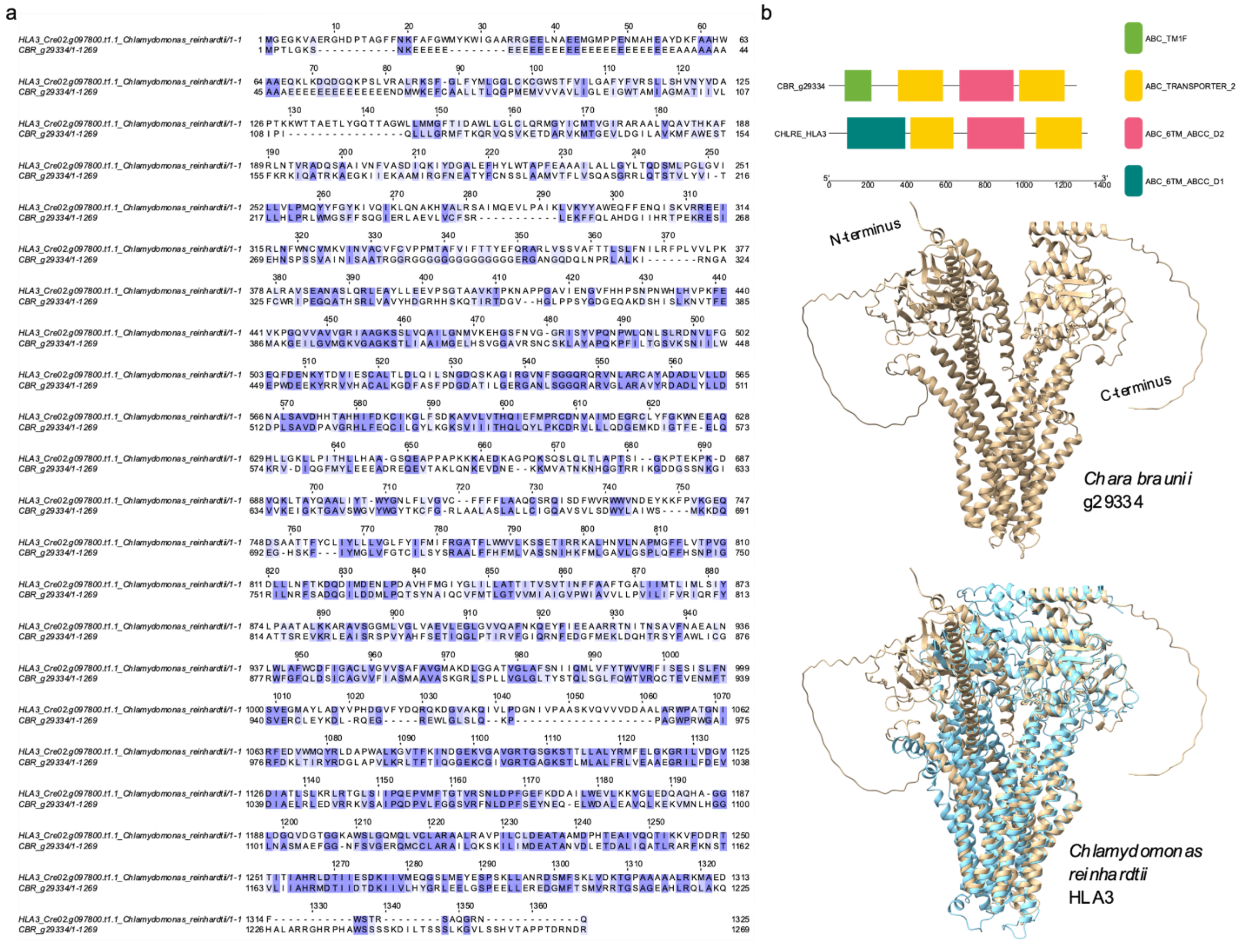

Fig. S10 Domain structure and alignment of HLA3 homolog. a. Sequence alignment of *Chara braunii* HLA3-homolog g29334 to *Chlamydomonas reinhardtii* HLA3 membrane protein. Alignment of M-coffee aligned amino acid sequences was generated using JalView v.2.11.2.7 (50% conservation colored blosum62 score). **b.** Comparative domain structure and structural render of *Chara braunii* HLA3-homolog (beige), in contrast to *Chlamydomonas reinhardtii* HLA3 (cyan). Domain structures were generated with InterPro data using TBtools II v.2.0.1.0, Protein models were generated via AlphaFold3 prediction (Abramson *et al.*, 2024) and visualized using ChimeraX v.1.6.1.

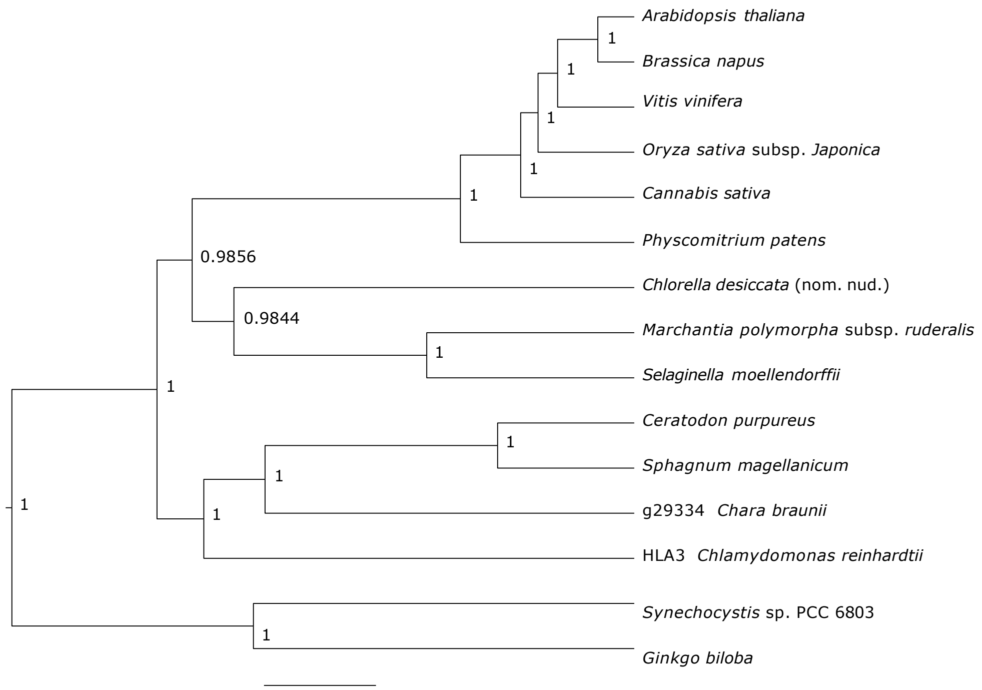

Fig. S11 Phylogenetic analysis of the *Chara braunii* g29334 gene product, a putative bicarbonate-transporting ATPase (HLA3) by Bayesian inference. Selected homologs from land plants and different green algae were aligned via M-Coffee and analyzed using BEAST 2. The depicted maximum clade credibility tree, generated via tree prior yule model and $1e^4$ logged MCMC chain length of $1e^7$ with 50% burnin, was built using TreeAnnotator a posterior probability limit of 0.5 for median node heights. The generated dendrogram was visualized using FigTree with posterior probabilities labeled on their respective nodes. A protein from *Ginkgo biloba* and one from the cyanobacterium *Synechocystis* sp. PCC 6803 were used as outgroups.

```
                             10        20        30        40        50        60        70        80
                    ....|....|....|....|....|....|....|....|....|....|....|....|....|....|....|....|
CBR_g15996          NIGSRPSKRTTMNDVQSLRAIPWVFAWTQTKLHLPVWLGIGTALRHVMEQKPENLQKLQQMHSEWPFFRATLDLIEMILA
CBR_g51453          NIGSRPTRRGKSGGIHQLRAIPWIFAWTQTRFILPAWLGVGEGLKAMFEKG--HKEELQEMYREWPFFQSTMDLVEMIFA
CBR_g8806           NIGSRPSKRRTDQGIGSLRAIPWVFAWTQTRIHLPVWLGVGAALKYAIEKDPANLDVLRQMHRGWPFFRVTLDLIEMVFA
CBR_g38936          NIGSRPSKRSTQGGITSLRAIPWVFAWTQTRVHLPVWLGVGTAIERLLEEDPSNLEVLRQMHREWPFFRVTLDLIEMVFA
CBR_g8801           NIGSRPSKRRSDLGIESLRAIPWVFAWTQTRIHLPVWLGVGAALKYAIEKDPANLDILRRMHSEWPFFRVTLDLVEMVFA
Zm P04711.2         NIGSRPAKRRPGGGITTLRAIPWIFSWTQTRFHLPVWLGVGAAFKFAIDKDVRNFQVLKEMYNEWPFFRVTLDLLEMVFA  C4 PEPC
Zm XP008676434      NIGSRPSKRKPSGGIESLRAIPWIFAWTQTRFHLPVWLGFGAAIKHIMQKDIRNIHILKEMYNEWPFFRVTLDLLEMVFA
Zm PWZ12751.1       NIGSRPSKRKPSGGIDSLRAIPWIFAWTQTRFHLPVWLGFGAAFKHVLQKDIRNLHMLQEMYNEWPFFRVTIDLVEMVFA
Zm AQK59537.1       NIGSRPSKRKPSGGIESLRAIPWIFAWTQTRFHLPVWLGFGAAIKHIMQKDIRNIHILKEMYNEWPFFRVTLDLLEMVFA
Zm NP001349349      NIGSRPSKRKPSAGIESLRAIPWIFAWTQTRFHLPVWLGFGAAFKHVLDKDIRNLQTLQEMYNQWPFFRVTIDLVEMVFA
Zm ONM54187.1       NIGSRPSKRKPSGGIDSLRAIPWIFAWTQTRFHLPVWLGFGAAFKNVLQKDIRNLHMLQEMYNEWPFFRVTIDLVEMVFA
Fp CAA88829.1       NIGSRPSKRKPSGGIESLRAIPWIFAWTQTRFHLPVWLGFGAAFKHAIKKDSKNLQMLQEMYKTWPFFRVTIDLVEMVFA
Fp pdb|3ZGB|A       NIGSRPSKRKPSGGIESLRAIPWIFAWTQTRFHLPVWLGFGAAFKHAIKKDSKNLQMLQEMYKTWPFFRVTIDLVEMVFA
Ft Q9FV66.1         NIGSRPSKRKPSGGIESLRAIPWIFAWTQTRFHLPVWLGFGAAFKQAIQKDSKNLQMLQEMYKTWPFFRVTIDLVEMVFA
Ft pdb|3ZGE|A       NIGSRPSKRKPSGGIESLRAIPWIFSWTQTRFHLPVWLGFGAAFKHAIQKDSKNLQMLQEMYKTWPFFRVTIDLVEMVFA  C4 PEPC
Ft pdb|4BXH|B       NIGSRPSKRKPSGGIESLRAIPWIFSWTQTRFHLPVWLGFGAAFKHAIQKDSKNLQMLQEMYKTWPFFRVTIDLVEMVFA  C4 PEPC
Ft Q9FV65.1         NIGSRPSKRKPSGGIESLRAIPWIFAWTQTRFHLPVWLGFGAAFKYAIEKDIKNLHMLQEMYKTWPFFRVTIDLVEMVFA
At OAP04148.1       NIGSRPSKRKPSGGIESLRAIPWIFAWTQTRFHLPVWLGFGAAFRYAIKKDVRNLHMLQDMYKQWPFFRVTIDLIEMVFA
At NP001031178      NIGSRPSKRKPSGGIESLRAIPWIFAWTQTRFHLPVWLGFGSAIRHVIEKDVRNLHMLQDMYQHWPFFRVTIDLIEMVFA
At pdb|8CJ5|A       NIGSRPSKRKPSGGIESLRAIPWIFAWTQTRFHLPVWLGFGSAIRHVIEKDVRNLHMLQDMYQHWPFFRVTIDLIEMVFA
At AAP43628.1       NIGSRPSKRKPSGGIESLRAIPWIFAWTQTRFHLPVWLGFGGAFKRVIQKDNKNLNMLKEMYNQWPFFRVTIDLVEMVFA

                             90       100       110       120       130       140       150       160
                    ....|....|....|....|....|....|....|....|....|....|....|....|....|....|....|....|
CBR_g15996          KADPRIFAMYDSLLAPKELSQFGHELRYKFQDMKELLLKVAGHKSFLENNTALRQRLILREPLMTPLHVLQALTLNKMRG
CBR_g51453          KADIHIAKHYDEVLVSPERRSLGEEMRKSFQRTIDMVLQVSGHQILAENNRTLLRKLICRTPYLNPINMLQVEIIRRLRG
CBR_g8806           KGDVRIAMLYEKMLVSEEFQTLGEEIRANFQNTRDLLLKVADIPRTLDNNPALKQRIILREPLTTPLNVQQVITLKKMRG
CBR_g38936          KGDIRIGELYDSLLVSDEYRAFGEELRRNYMKTRRLILEVASHRQTLDNNPAVKQNIIVRQPLMTPLNLQQAITLRRMRG
CBR_g8801           KGDPRIASMYDKILVPDELRSLGEEILHNYHLTRDLLLQVAGHSLTLDNNPALKQRIILREPLTTPLNVQQALTLKKMRG
Zm P04711.2         KGDPGIAGLYDELLVAEELKPFGKQLRDKYVETQQLLLQIAGHKDILEGDPFLKQGLVLRNPYITTLNVFQAYTLKRIRG  C4 PEPC
Zm XP008676434      KGDPGIAAVYDKLLVADDLQSFGEQLRKNYEETKELLLQVAGHKDVLEGDPYLKQRLRLRESYITTLNVCQAYTLKRIRG
Zm PWZ12751.1       KGNPGIAALYDKLLVSEELHPLGEKLRANYEETQKLLLQVAGHRDLLEGDLYLKQRLRLRDAYITTLNVCQAYTLKRIRG
Zm AQK59537.1       KGDPGIAAVYDKLLVADDLQSFGEQLRKNYEETKELLLQVAGHKDVLEGDPYLKQRLRLRESYITTLNVCQAYTLKRIRG
Zm NP001349349      KGDPGIAALYDKLLVSEDLWALGARLRANYEETKQLLLQVAGHKDLLEGDPYLKQRLRIRDSYTTALNVCQAYTLKRIRG
Zm ONM54187.1       KGNPGIAALYDKLLVSEELHPLGEKLRANYEETQKLLLQVAGHRDLLEGDLYLKQRLRLRDAYITTLNVCQAYTLKRIRG
Fp CAA88829.1       KGDPGIAALNDKLLVSEDLWPFGESLRANYEETKDYLLKIAGHRDLLEGDPYLKQRIRLRDSYITTLNVCQAYTLKRIRG
Fp pdb|3ZGB|A       KGDPGIAALNDKLLVSEDLWPFGESLRANYEETKDYLLKIAGHRDLLEGDPYLKQRIRLRDSYITTLNVCQAYTLKRIRG
Ft Q9FV66.1         KGDPGIAALNDKLLVSEDLWPFGESLRANYEETKDYLLKIAGHKDLLEGDPYLKQRLKLRDSYITTLNVCQAYTLKRTRG
Ft pdb|3ZGE|A       KGNPGIAALNDKLLVSEDLRPFGESLRANYEETKNYLLKIAGHKDLLEGDPYLKQGIRLRDPYITTLNVCQAYTLKRIRG  C4 PEPC
Ft pdb|4BXH|B       KGNPGIAALNDKLLVSEDLRPFGESLRANYEETKNYLLKIAGHKDLLEGDPYLKQGIRLRDPYITTLNVCQAYTLKRIRG  C4 PEPC
Ft Q9FV65.1         KGDPGIAALNDKLLVSEDLWSFGESLRANYEETKNLLLKIAGHKDLLEGDPYLRQRLRLRDSYITTLNVCQAYTLKRIRG
At OAP04148.1       KGDPGIAALYDKLLVSEDLWAFGEKLRANFDETKNLVLQTAGHKDLLEGDPYLKQRLRLRDSYITTLNVCQAYTLKRIRG
At NP001031178      KGDPGIAALYDKLLVSEELWPFGEKLRANFEETKKLILQTAGHKDLLEGDPYLKQRLRLRDSYITTLNVCQAYTLKRIRG
At pdb|8CJ5|A       KGDPGIAALYDKLLVSEELWPFGEKLRANFEETKKLILQTAGHKDLLEGDPYLKQRLRLRDSYITTLNVCQAYTLKRIRG
At AAP43628.1       KGDPGIAALYDRLLVSEELQPFGEQLRVNYQETRRLLLQVAGHKDILEGDPYLRQRLQLRDPYITTLNVCQAYTLKQIRG
```

Fig. S12 Partial sequence alignment of *C. braunii* PEP carboxylases (PEPC) and related plant enzymes. Protein sequences of putative *C. braunii* (Cbr) PEP carboxylases were aligned with PEP carboxylase sequences from C3 plants such as *Arabidopsis thaliana* (At) and *Flaveria pringlii* as well as C4 plants such as *Zea mays* (Zm) and *Flaveria trinervia* (Ft). PEPC isoforms proven to be involved in the C4 plant CCM are marked as C4 PEPC (right side). Specific amino acid exchanges known to change the biochemistry of C4 PEPC isoforms (Paulus et al., 2013) at position 26 (A to S) and position 136 (R to G) are marked by red arrows.

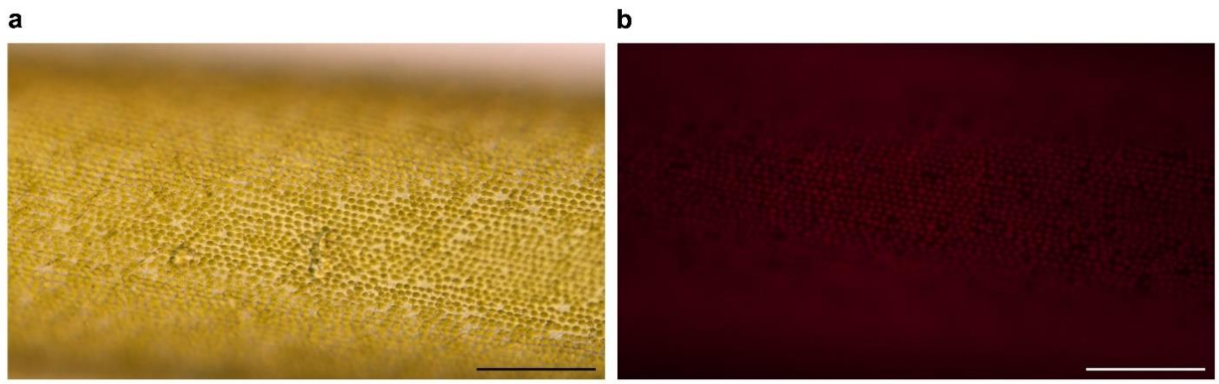

Fig. S13 Stationary chloroplasts in internodal cells of *Chara braunii*. a. Brightfield image of an internodal cell section and **b.** chlorophyll autofluorescence image showing rows of immobile chloroplasts (Olympus BX-51, 40x objective). Scale bars are 50 µm.

SI References

Abramson J, Adler J, Dunger J et al. 2024. Accurate structure prediction of biomolecular interactions with AlphaFold 3. *Nature* 630: 493–500.

Batut B, van den Beek M, Doyle MA, Soranzo N. 2021. RNA-Seq Data Analysis in Galaxy RNA Bioinformatics. *Methods in Molecular Biology.* In: E Picardi, eds. New York, NY: Humana, 2284

Batut B, Freeberg M, Heydarian M, Erxleben A, Videm P, Blank C, Doyle M, Soranzo N, van Heusden P, Delisle L. 2022. Reference-based RNA-Seq data analysis. at https://training.galaxyproject.org/training-material/topics/transcriptomics/tutorials/ref-based/tutorial.html

The Galaxy Community. 2022. The Galaxy platform for accessible, reproducible and collaborative biomedical analyses. *Nucleic Acids Research* **50**: W345–W351.
